# Heterozygosity and homozygosity regions affect reproductive success and the loss of reproduction: A case study with litter traits in pigs

**DOI:** 10.1016/j.csbj.2022.07.039

**Published:** 2022-07-26

**Authors:** Zitao Chen, Zhenyang Zhang, Zhen Wang, Zhe Zhang, Qishan Wang, Yuchun Pan

**Affiliations:** a Department of Animal Science, College of Animal Science, Zhejiang University, 866# Yuhangtang Road, Hangzhou East 310058, China; b Hainan Institute, Zhejiang University, Yongyou Industry Park, Yazhou Bay Sci-Tech City, Sanya South 572000, China

**Keywords:** Association analysis, Mortality, Reproductive success, Runs of heterozygosity, Runs of homozygosity

## Abstract

Runs of heterozygosity (ROHet) and homozygosity (ROH) harbor useful information related to traits of interest. There is a lack of investigating the effect of ROHet and ROH on reproductive success and the loss of reproduction in mammals. Here, we detected and characterized the ROHet and ROH patterns in the genomes of Chinese indigenous pigs (i.e., Jinhua, Chun’an, Longyou Black, and Shengxian Spotted pigs), revealing the similar genetic characteristics of indigenous pigs. Later, we highlighted the underlying litter traits-related ROHet and ROH using association analysis with linear model in these four indigenous pig breeds. To pinpoint the promising candidate genes associated with litter traits, we further in-depth explore the selection patterns of other five pig breeds (i.e., Erhualian, Meishan, Minzhu, Rongchang, and Diqing pigs) with different levels of reproduction performance at the underlying litter traits-related ROHet and ROH using *F*_ST_ and genetic diversity ratio. Then, we identified a set of known and novel candidate genes associated with reproductive performance in pigs. For the novel candidate genes (i.e., *CCDC91*, *SASH1*, *SAMD5*, *MACF1*, *MFSD2A*, *EPC2*, and *MBD5*), we obtained public available datasets and performed multi-omics analyses integrating transcriptome-wide association studies and comparative single-cell RNA-seq analyses to uncover the roles of them in mammalian reproductive performance. The genes have not been widely reported to be fertility-related genes and can be complementally considered as prior biological information to modify genomic selections models that benefits pig genetic improvement of litter traits. Besides, our findings provide new insights into the function of ROHet and ROH in mammals.

## Graphical abstract

**Figure:**
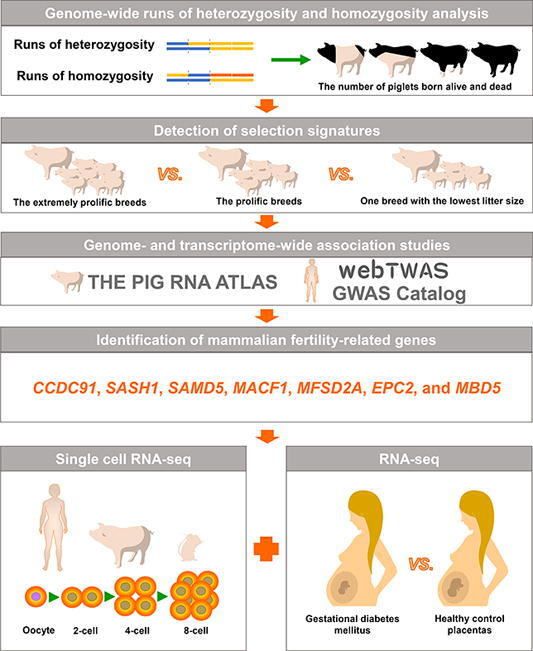

## 1 Introduction

Litter traits are one of the most important traits for breeding in pig production [1], [2]. Improving total number born (**TNB**) and number born alive (**NBA**) traits are of great interest as enhancing the economic profit, and become the main breeding goals in the modern pig industry [1], [3]. Meanwhile, sow reproductive performances are limited by embryonic mortality and fetal losses during the pregnancy period [4]. Recently, the genetic mechanisms of the loss of reproduction have gradually received attention, and a few studies have explored underlying genetic architecture of the number of piglets born dead traits in pigs [3], [5], [6]. Moreover, the extreme litter sizes reduce welfare and survival of the piglets at weaning, and decreasing the variation in litter size will lead to more sustainable breeding in terms of increasing piglet survival [7]. Generally, litter traits have low heritability [8], [9], therefore, genetic analyses should be systematically conducted for the traits to generate the in-depth biological knowledge that can significantly improve the selection efficiency of genomic selection and benefit genetic improvements of low heritability traits [10], [11]. In addition, swine can be used as an alternative animal model, which likely overcome the limitations in human and other models, one reason is that swine are highly similar to humans in many aspects, e.g., hormonal cycle and physiology [12]. For this reason, pig is considered as the larger animal model for human infertility in the field of reproduction research [13].

The rapid development of high-throughput sequencing has opened up the possibility of characterizing the genome in terms of homozygosity or heterozygosity. Runs of heterozygosity (**ROHet**), known as heterozygosity-rich regions, emerged as a more recent concept, and have been used to identify genomic regions that are under gene introgression or admixture [14], [15]. ROHet avoid the deleterious effects of harmful homozygous genotype aggregation on specific traits and maintain heterozygote advantage at immune-related genes [16], benefiting reproduction performance. In the meantime, runs of homozygosity (**ROH**) have been a helpful strategy to detect regions under selection [17]. A higher incidence of ROH could lead to congenital anomalies [18], resulting in the loss of reproduction. Previous studies mainly used the distribution of ROHet or ROH and the function of genes within ROHet or ROH to investigate the population characteristics [19], [20]. Based on this, association analyses using ROHet and ROH provide a powerful strategy to identify genomic regions associated with traits of interest in human [21] and livestock [16].

China has the most genetic resources of domestic pigs [22], [23]. Jinhua (**JH**), Chun’an (**CA**), Longyou Black (**LY**), and Shengxian Spotted (**SX**) pigs originated in eastern China and have similar characteristics to other indigenous breeds, e.g., improved meat quality, disease resistance, and high fertility in comparison with intensively-reared commercial pig breeds (e.g., Large white, Landrace, and Duroc pig breeds) [24], [25], [26]. These excellent characteristics mark them as the best material to uncover the genetic mechanism of economic traits and support the sustainable development of the pig industry [27]. It is well reported that Chinese indigenous pigs have excellent litter sizes and contribute to the improvement of fertility in intensively-reared commercial pig breeds [28], [29]. However, there is a lack of investigating the effect of ROHet and ROH on litter traits in Chinese indigenous pigs.

In this study, we performed integrated genomic analyses to explore the genetic mechanisms of reproductive success and the loss of reproduction in mammal. Firstly, we systematically investigated the distribution of ROHet and ROH in the genomes of indigenous pigs to detect and characterize the ROHet and ROH patterns, as well as to reveal ROHet and ROH islands that contain the candidate genes related to breed-specific traits of Chinese indigenous pig breeds. Then, we conducted association analyses to identify the litter traits-related regions, and uncovered the effects of the identified regions and promising candidate genes on reproductive performance in other pig breeds via traits-specific selection signatures. Furthermore, we performed comparative transcriptome analysis to identify important fertility-related genes in human, pig, and mouse.

## 2 Materials and methods

### 2.1 Ethics statement

Animal care and experiments were conducted in accordance with the Chinese guidelines for animal welfare and were approved by the Animal Care and Use Committee of Zhejiang University, Hangzhou, China (permit number: ZJU20160346).

### 2.2 Animal resources, phenotypes, and SNP genotyping

Blood samples of JH (N = 193: 41 sires and 152 dams), CA (N = 98: 31 sires and 67 dams), LY (N = 94: 32 sires and 62 dams), and SX (N = 174: 43 sires and 131 dams) were collected from the national or provincial conservation farms in Zhejiang province of China. We collected all sires and dams except for full siblings according to the pedigree records in conservation farms, thus these individuals represent the most comprehensive genetic diversity of each pig breed. Litter traits included TNB, NBA, and total number of piglets born dead (**TND**). Herein, NBA was identified as the piglets alive after the farrowing; TND included the number of stillborn piglets and the mummified piglets at birth. We removed records if TNB was equal to zero, or if only one record per sow was available. After data editing, 4742 litter records from 412 sows were available. After grouping records according to sow parity, the farrowing records were 1236, 624, 537, 507, 645, 543, 489, and 161 for parity groups 1, 2, 3, 4, 5, 6, 7, and 8, respectively. The descriptive statistics of TNB, NBA, and TND per parity were listed in Supplementary Table 1.

To get a better understanding of the genetic architecture of litter size variability traits, we obtained log-transformed variance of residuals of TNB (**LnVarTNB**), NBA (**LnVarNBA**), and TND (**LnVarTND**) traits from the records of each traits following [9], [30]: firstly, we used the formula (1) to calculate a residual for available observation for TNB, NBA, and TND traits; secondly, a variance of these residuals was log-transformed to generate a unique value of LnVar for each of TNB, NBA, and TND traits per sow.

(1) y=Wα+Zb+Upe+e

where y is the TNB, NBA, and TND of the individuals; *α* is a vector of fixed effects, including farm-year-season of the farrowing (64 levels) and parity of the sow (8 levels); *b* is a vector of additive genetic effects and is set as *b* ∼ *N*(0, G $\sigma_{b}^{2}$), where G is the genomic relationship matrix [31]; *pe* is a vector of permanent environmental sow effects and is set as *pe* ∼ *N*(0, I $\sigma_{\mathrm{pe}}^{2}$); W, Z, and U are incidence matrices for *α*, *b*, and *pe*; *e* is a vector of residuals and is set as *e* ∼ *N*(0, I $\sigma_{e}^{2}$); I is an identity matrix.

Genomic DNA was extracted from blood samples with a standard phenol–chloroform method and genotyped using the GeneSeek Genomic Profiler Porcine SNP BeadChip (Neogen Corporation, Lansing, MI, USA), which contains 50,697 SNPs. Subsequently, quality control was performed using PLINK (v1.9) software [32], of which SNPs were filtered out with a call rate less than 90 % [33]. A final set of 44,901 informative SNPs were used for further ROHet and ROH analysis.

Furthermore, we obtained whole-genome re-sequencing (**WGS**) data of five pig breeds (Erhualian, **EHL**, N = 12; Minzhu, **MZ**, N = 10; Meishan, **MS**, N = 14; Rongchang, **RC**, N = 12; Diqing, **DQ**, N = 12) from the NCBI SRA database to uncover the evolutionary history of the regions and potential effects on litter traits in pigs (Supplementary Table 2). These pig breeds contained two extremely prolific breeds (EHL and MS), two prolific breeds (MZ and RC), and one breed with the lowest litter size (DQ). Herein, raw reads were filtered using fastp software with default parameters. Filtered reads were aligned to the latest pig reference genome (*Sscrofa*11.1) using the Burrows-Wheeler alignment (v0.7.17-r1188) tool [34] with the parameters for paired-end reads. Subsequently, SAM files were merged and sorted using SAMtools (v1.9) software [35]. SNP calling for each individual was implemented using the HaplotypeCaller program and further joint calling using GenotypeGVCFs program of GATK4 (v4.1.6.0) software [36]. We filtered SNPs using VariantFiltration program with the parameters: --filter-expression “QD less than 2.0 || FS > 60.0 || MQ less than 40.0 || SOR > 3.0 || MQRankSum < –12.5 || ReadPosRankSum < –8.0.” Quality control was performed using PLINK (v1.9) software [32], of which SNPs were filtered out with a call rate less than 90 % and minor allele frequency (MAF) less than 0.01. The remaining 34,265,460 informative SNPS were used for signatures of selection detection. In addition, the performance of TNB, NBA, and TND traits for each available breed was obtain from *Animal Genetic Resources in China: Pigs*.

### 2.3 Runs of heterozygosity and homozygosity detection

ROHet and ROH were detected separately for each individual using detectRUNS R package v0.9.6. A ROHet was identified based on a consecutive strategy [37] with the following parameters: (i) a minimum ROHet length of 1 Mb; (ii) a minimum number of SNPs within a ROHet>15; (iii) no homozygous and missing genotypes; (iv) the maximum gap of 500 kb between consecutive heterozygous SNPs. In addition, a ROH was defined based on a sliding window strategy [32] with the following parameters: (i) a sliding window of 50 SNPs; (ii) a minimum ROH length of 1 Mb; (iii) no heterozygous and missing genotypes; (iv) the maximum gap of 500 kb between consecutive homozygous SNPs; (v) a minimum SNP density of two SNP per Mb; (vi) a minimum number of SNPs within a ROH at least 85, 74, 50, and 84 in JH, CA, LY, and SX pigs, which were computed by the formula (2) [38]. Next, we filtered out the ROHet and ROH with less than five SNPs and present in less than 5 % of the individuals.

(2) $$l=\;\frac{\ln\frac{\alpha }{n_{s}\times n_{i}}}{ln\left(1-\overset{¯}{\mathrm{het}}\right)}$$

where *α* is the percentage of false positive ROH (set to 0.05), *n_s_* is the number of SNPs per individual, *n_i_* is the number of individuals, and *het* is the proportion of heterozygosity across all SNPs.

### 2.4 Rohet and ROH size categories

We categorized the ROHet and ROH for each breed into five length classes (1–2 Mb, 2–4 Mb, 4–8 Mb, 8–16 Mb, and > 16 Mb). In addition, we estimated ROH-based inbreeding coefficients (***F*_ROH_**) using the formula (3) [39].

(3) $$F_{\mathrm{ROH}}=\;\frac{\sum L_{\mathrm{ROH}}}{L_{\mathrm{AUTO}}}$$

where $\sum L_{\mathrm{ROH}}$ is the total length of all the ROH detected in an individual, $L_{\mathrm{AUTO}}$ is the total length of the autosomes covered by SNPs (2.452 Gb in this study).

Furthermore, we generated the false discovery rate (**FDR**) adjusted empirical *P*-values [40], [41] by genome-wide ranking of the percentage of SNP occurrences to identify highly heterozygous regions (**ROHet island**) or highly homozygous regions (**ROH island**) via extracting SNPs with the FDR adjusted empirical *P*-values less than 0.01 located in a ROHet or ROH.

### 2.5 Genome-wide ROHet and ROH analysis

We fitted linear model to investigate the association between unique heterozygous or homozygous regions (presence or absence) and six reproductions traits using *lm* function in R v4.0.5 [42] via the formula (4).

(4) $$y_{\mathrm{jkli}}={\mathrm{breed}}_{j}+\;{\mathrm{farmyearseason}}_{k}+{\mathrm{region}}_{l}+e_{\mathrm{jkli}}$$

where $y_{\mathrm{jkli}}$ is the phenotype of the i individual, i.e., TNB, NBA, and TND per parity, LnVarTNB, LnVarNBA, and LnVarTND; ${\mathrm{breed}}_{j}$ is the fixed effect of the j breed; ${\mathrm{farmyearseason}}_{k}$ is the fixed effect of the k farm-year-season of the farrowing; ${\mathrm{region}}_{l}$ is the fixed effect with two levels of ROHet or ROH (presence as one and absence as zero); $e_{\mathrm{ijkl}}$ is the random residual. Here, we used the ROHet (N = 95) and ROH (N = 203) detected in>1 % of the individuals that had phenotypic records. Then, the Bonferroni correction was applied to determine the threshold of ROHet or ROH association analysis. ROHet or ROH with *P*-values lower or equal than 0.05/N, were regarded as genome-wide significant ROHet or ROH. ROHet or ROH with a *P*-value higher than 0.05/N but lower than 1/N were considered as genome-wide suggestive significant ROHet or ROH.

### 2.6 Signatures of selection detection

We investigated selection signatures across the highest frequency ROHet and ROH using VCFtools version 0.1.14 [43] via *F*_ST_ [44] and genetic diversity ratio (**θ_π_ statistic**). We calculated θ_π_ statistic across the highest frequency ROHet and ROH regions in JH, CA, LY, and SX pigs to measure the variability of polymorphism levels across the regions between these pigs. To get more comprehensive results of genetic differentiation among the extremely prolific breeds, prolific breeds and breeds with the lowest litter size, five pig breeds (EHL, MZ, MS, RC, and DQ) were further compared with each other using *F*_ST_ and θ_π_ statistic with the use of a sliding window method (50 kb window and 10 kb step). The θ_π_ statistic between two breeds was calculated as ln(θ_π|breed1_/θ_π|breed2_). Moreover, we divided five pig breeds into two groups (group 1: MS, RC, and DQ; group 2: EHL, MZ, and DQ) and separately conducted *F*_ST_ analysis. Here, we uncovered the selection patterns of the highest frequency ROHet and ROH with the breeds of group 1, then the breeds of group 2 were further used to check the patterns.

### 2.7 Gene annotation

Genome annotations were based on *Sscrofa*11.1, genes overlapping with ROHet and ROH islands were treated as candidate genes. We downloaded the information of tissue-specific and human-pig homologous genes from the Pig RNA Atlas [45]. Furthermore, we collected the results of genome-wide association studies (**GWAS**) summary statistic and transcriptome-wide association studies (**TWAS**) research for fertility-related traits in human from the webTWAS database [46] to further explore the potential roles of the novel genes in female mammalian reproductive function.

### 2.8 RNA-seq data processing and bioinformatics analysis

The single cell RNA-seq (**scRNA-seq**) data of in vivo embryos (oocyte, 2-cell, 4-cell, and 8-cell) from human, mouse [47], and pig [48] were collected from the GEO public database (GSE44183 and GSE139512) to conduct comparative transcriptome analysis. Furthermore, the RNA-seq data of gestational diabetes mellitus (**GDM**) and healthy control placentas was collected from the database (GSE154414). We downloaded the datasets and further processed that using FastQC (v0.11.4) software to obtain clean reads with default parameters. Clean reads were then mapped to the reference genome and annotated transcripts (human: GRCh38; mouse: GRCm39; pig: *Sscrofa*11.1) by Hisat2 (v2.1.0) software [49]. The read numbers mapped to each gene were counted and quantified as fragments per kilobase million mapped reads (**FPKM**) using featureCounts (v2.0.3) software [50]. To reduce the bias of genes and gene expression levels potentially affected by different library preparation and sequencing platforms, we retained the transcripts with FPKM larger than zero.

### 2.9 Functional enrichment analyses

In addition, all ROHet and ROH islands were annotated using the Animal QTL Database (Hu et al., 2019). We conducted QTL enrichment analyses using GALLO (Fonseca et al., 2020). We used ClusterProfiler package [51] to perform Gene ontology (**GO**) and Kyoto Encyclopedia of Genes and Genomes (**KEGG**) pathway enrichment analyses. Here, QTLs and terms with the FDR less than 0.05 were retained.

## 3 Results

### 3.1 Runs of heterozygosity detection and annotation

**Fig. 1:**
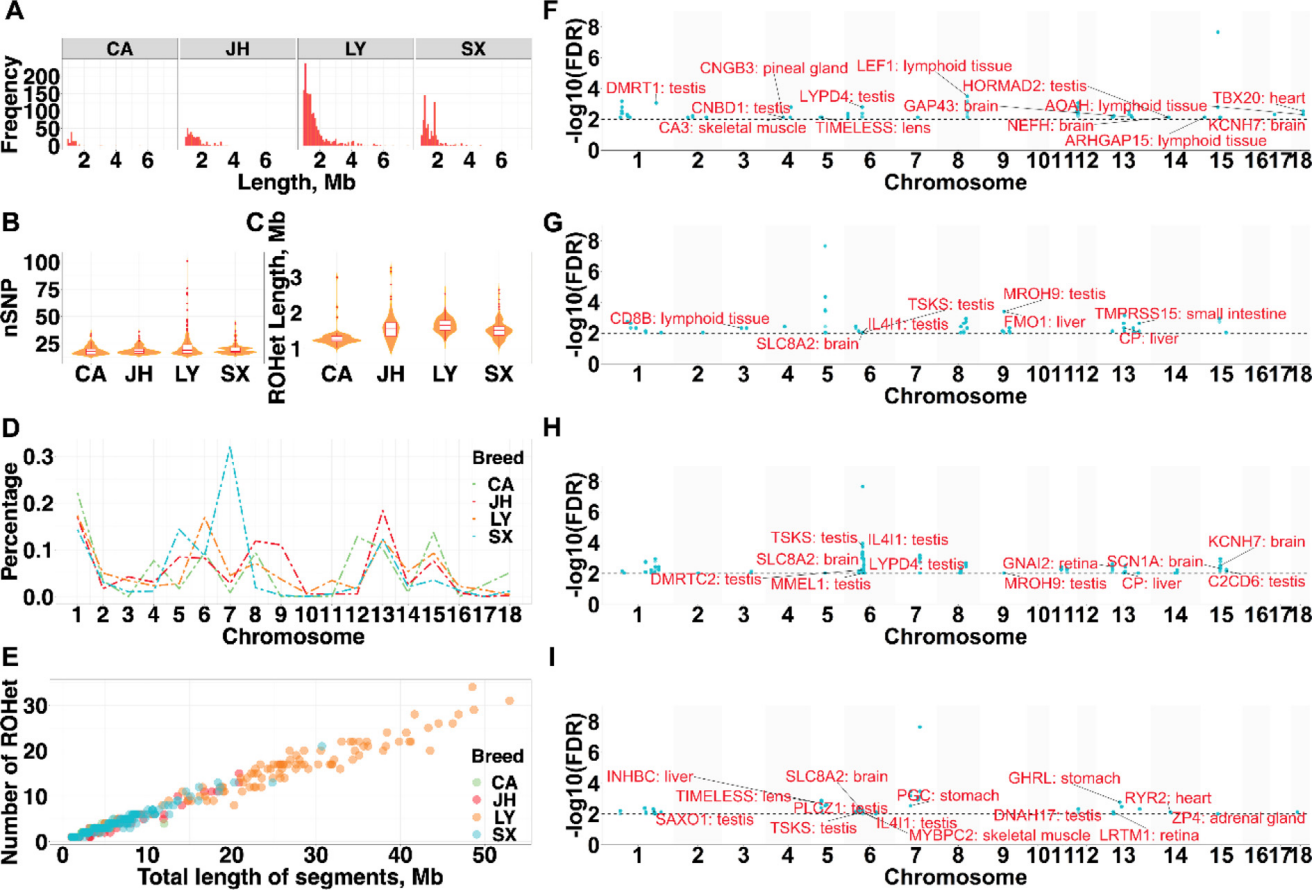
ROHet in the genomes of Chun’an (CA), Jinhua (JH), Longyou Black (LY), and Shengxian Spotted (SX) pig populations. (A) Number of ROHet; (B) Average number of SNPs in ROHet; (C) Average ROHet length; (D) Number of total ROHet per chromosome; (E) Number and total length of ROHet per individual; Manhattan plot of the incidence of heterozygotes within ROHet in CA (F), JH (G), LY (H), and SX (I) populations.

In total, we detected 117, 353, 1,505, and 676 ROHet in the CA, JH, LY, and SX populations, with an average of 1.19, 1.83, 16.01, and 3.89 ROHet for each pig breed, respectively (Fig. 1A). The average number of SNPs in each ROHet was 18.68 ± 4.11, 18.69 ± 3.55, 21.09 ± 7.33, and 19.57 ± 4.66, and the mean length of ROHet was 1.35 ± 0.55, 1.58 ± 0.55, 1.67 ± 0.84, and 1.55 ± 0.51 Mb in the CA, JH, LY, and SX populations (Fig. 1B, C). Further, we observed that a higher number of ROHet on *sus scrofa* chromosome (**SSC**) 1 and 13, which had the longest chromosome length. To note, the SX population had the highest number of ROHet on SSC 7, showing an extremely different pattern with other populations (Fig. 1D). The short fragment (1–2 Mb) of ROHet accounted for the largest fraction of ROHet, and no ROHet with the length>8 Mb was observed (Table 1). Meanwhile, the number of ROHet decreased with the increasing of ROHet length (Fig. 1E).

**Table 1:** Number, percentage, and average length for ROHet categories.

Categories	Jinhua			Chun'an			Longyou Black			Shengxian Spotted		
	Count	Percentage (%)	Average (Mb)	Count	Percentage (%)	Average (Mb)	Count	Percentage (%)	Average (Mb)	Count	Percentage (%)	Average (Mb)
ROHet: 1-2 Mb	301	85.27	1.39	113	96.58	1.26	1219	81	1.34	625	92.46	1.44
ROHet: 2-4 Mb	52	14.73	2.67	2	1.71	3.08	228	15.15	2.66	48	7.1	2.74
ROHet: 4-8 Mb	–	–	–	2	1.71	4.75	58	3.85	4.68	3	0.44	4.51
ROH: 1-2 Mb	55	0.73	1.81	20	0.98	1.64	171	11.25	1.64	28	0.46	1.86
ROH: 2-4 Mb	1205	15.94	3.23	419	20.55	3.06	563	37.04	2.95	726	11.84	3.18
ROH: 4-8 Mb	2870	37.97	5.69	687	33.69	5.77	417	27.43	5.71	1999	32.63	5.9
ROH: 8-16 Mb	1960	25.93	11.22	528	25.89	11.08	236	15.53	10.65	1685	27.5	11.38
ROH: > 16 Mb	1469	19.43	33.54	385	18.89	35.95	133	8.75	31.79	1689	27.57	35.45

To explore the function of ROHet islands, we conducted tissue-specific genes annotation and QTL enrichment analysis (Fig. 1F**-I**). For CA pigs, 17 out of 167 candidate genes were specifically expressed in a single tissue. The genome regions overlapping with ROHet islands were mostly significantly related to days to 100 kg, carcass weight (cold), umbilical hernia, backfat at last rib, and shear force at first peak traits (Supplementary Fig. 1A). For JH pigs, a total of 137 candidate genes were located in ROHet islands, and involved in production (e.g., days to 100 kg), meat and carcass (e.g., intramuscular fat content), and health (e.g., actinobacillus pleuropneumoniae susceptibility) traits (Supplementary Fig. 1**B**). For LY pigs, we detected 179 candidate genes, of which had 12 tissue-specific genes. These genes were linked with basophil number, intramuscular fat content, days to 100 kg, blood non-esterified fatty acid level, and fat androstenone level traits (Supplementary Fig. 1**C**). For SX pigs, we detected 146 candidate genes, which were associated with average backfat thickness, meat color a*, shear force at first peak, fat androstenone level, and backfat at last rib traits (Supplementary Fig. 1**D**). Furthermore, we found that there were 66 ROHet shared by at least two pig breeds, and 41, 99, 658, and 116 unique ROHet detected in the CA, JH, LY, and SX populations, respectively (Supplementary Fig. 2**A**).

### 3.2 Runs of homozygosity detection and annotation

**Fig. 2:**
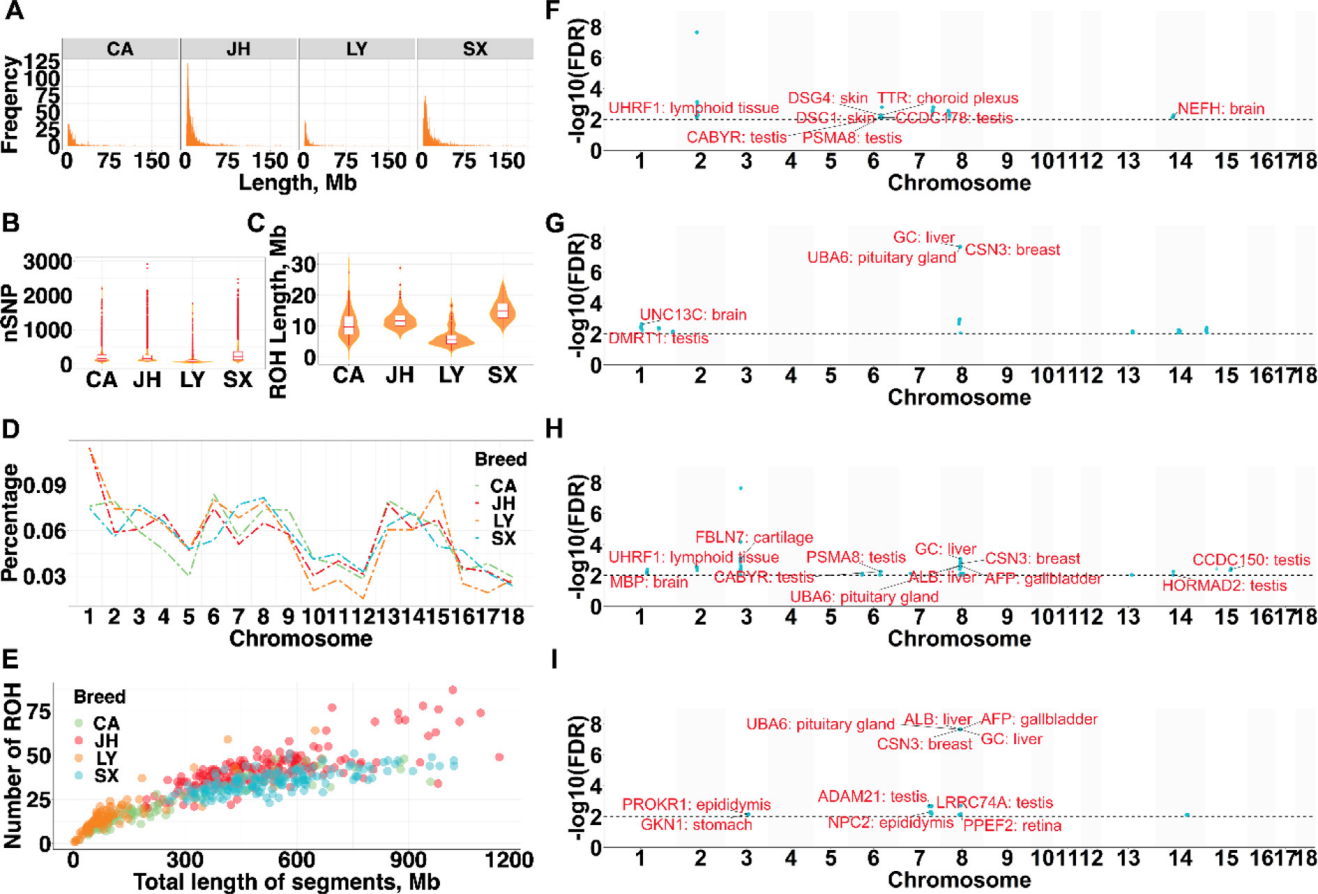
ROH in the genomes of Chun’an (CA), Jinhua (JH), Longyou Black (LY), and Shengxian Spotted (SX) pig populations. (A) Number of ROH; (B) Average number of SNPs in ROH; (C) Average ROH length; (D) Number of total ROH per chromosome; (E) Number and total length of ROH per individual; Manhattan plot of the incidence of homozygous within ROH in CA (F), JH (G), LY (H), and SX (I) populations.

In total, we identified 17,245 ROH in the 559 individuals considered (Table 1). In terms of average number of ROH per individual, the JH population had the largest number (39.17 ROH per animal), followed by the SX (35.21 ROH), CA (20.81 ROH), and LY (16.17 ROH) populations (Fig. 2A). The average number of SNPs in each ROH was 238.66 ± 241.91, 230.93 ± 228.94, 132.84 ± 173.59, and 300.21 ± 275.92 for the CA, JH, LY, and SX populations (Fig. 2B). Meanwhile, the LY population had the lowest average value (7.28 Mb) of the total ROH length, and the SX, CA, and JH populations were 15.21, 12.25, and 12.12 Mb, respectively (Fig. 2C). Further, we found that the patterns of the number of ROH on each chromosome were mostly similar in the four populations considered, whereas the SX population showed slightly different trends on SSC 7, 15, 16 (Fig. 2D). In addition, the number and length of ROH had negative correlations (Fig. 2E). The *F*_ROH_ showed that the SX and JH populations had the highest level of inbreeding (0.24 and 0.21, respectively), followed by the CA (0.11) and LY populations (0.05) (Supplementary Table 3). Nevertheless, we observed the similar trends of the *F*_ROH_ on each chromosome in the studied populations, except for the LY population (Supplementary Fig. 3).

There were 136 ROH shared by at least two pig breeds, and 1372, 5355, 1184, and 4112 unique ROH detected in the CA, JH, LY, and SX populations, respectively (Supplementary Fig. 2**B**). We found several shared candidate genes overlapping with the ROH islands of the studies breeds, e.g., *UHRF1*, *UBA6*, *GC*, *AFP* and *CHN3*. In addition, the QTLs overlapping with the ROH islands were mostly related to meat and carcass traits (e.g., backfat at last rib, intramuscular fat content, and fat androstenone level traits). Nevertheless, we found some QTLs associated with exterior traits (e.g., ear weight and thoracic vertebra number traits) reached the significant threshold (Supplementary Fig. 4).

### 3.3 Litter traits-related ROHet and ROH detection

**Fig. 3:**
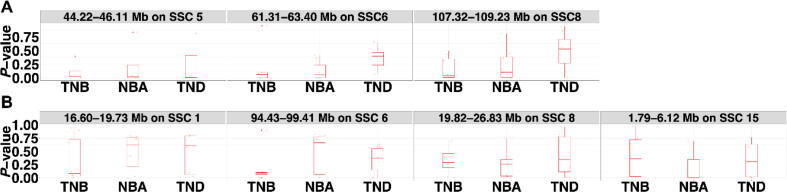
The association degree between the highest frequency (A) ROHet or (B) ROH and litter traits among parties.

The association analysis revealed 17, 7, 14, and 1 genome-wide significant ROHet for TNB, NBA, TND, and LnVarNBA traits, and 37, 39, 16, 4, and 2 ROHet reached the suggestive significance level for TNB, NBA, TND, LnVarNBA, and LnVarTND traits, respectively (Supplementary Table 4). Later, we counted the genome-wide significant ROHet and the ROHet reached the suggestive significance level, 44.22–46.11 Mb on SSC 5, 61.31–63.40 Mb on SSC 6, and 107.32–109.23 Mb on SSC 8 had the highest frequency (Fig. 3A). Herein, 13 out of 40 promising candidate genes were located on the highest frequency ROHet and overlapped with ROHet islands completely or partly, e.g., *CAMK2D*, *UGT8*, *ARSJ*, and *OVCH1* (Supplementary Table 5).

Meanwhile, we identified 7, 6, 17, 1, and 2 genome-wide significant ROH for TNB, NBA, TND, LnVarTNB, and LnVarNBA traits. There were 40, 23, 37, 6, 2, and 1 ROH reached the suggestive significance level for TNB, NBA, TND, LnVarTNB, LnVarNBA, and LnVarTND traits (Supplementary Table 6). In addition, 94.43–99.41 Mb on SSC 6 got the highest frequency, followed by 16.60–19.73 Mb on SSC 1, 19.82–26.83 Mb on SSC 8, and 1.79–6.12 Mb on SSC 15 (Fig. 3B). We found 5 out of 63 promising candidate genes were located on the highest frequency ROH and ROH islands, i.e., *EPC2*, *MMADHC*, *MBD5*, *KIF5C*, and *ACVR2A* (Supplementary Table 7).

Furthermore, we observed that the different association degree between the highest frequency ROHet (or ROH) and litter traits among parities (Fig. 3), which revealed that the genomic structures of piglets born alive and dead were different among parity groups.

### 3.4 Pattern of selection at the highest frequency litter traits-related ROHet

**Fig. 4:**
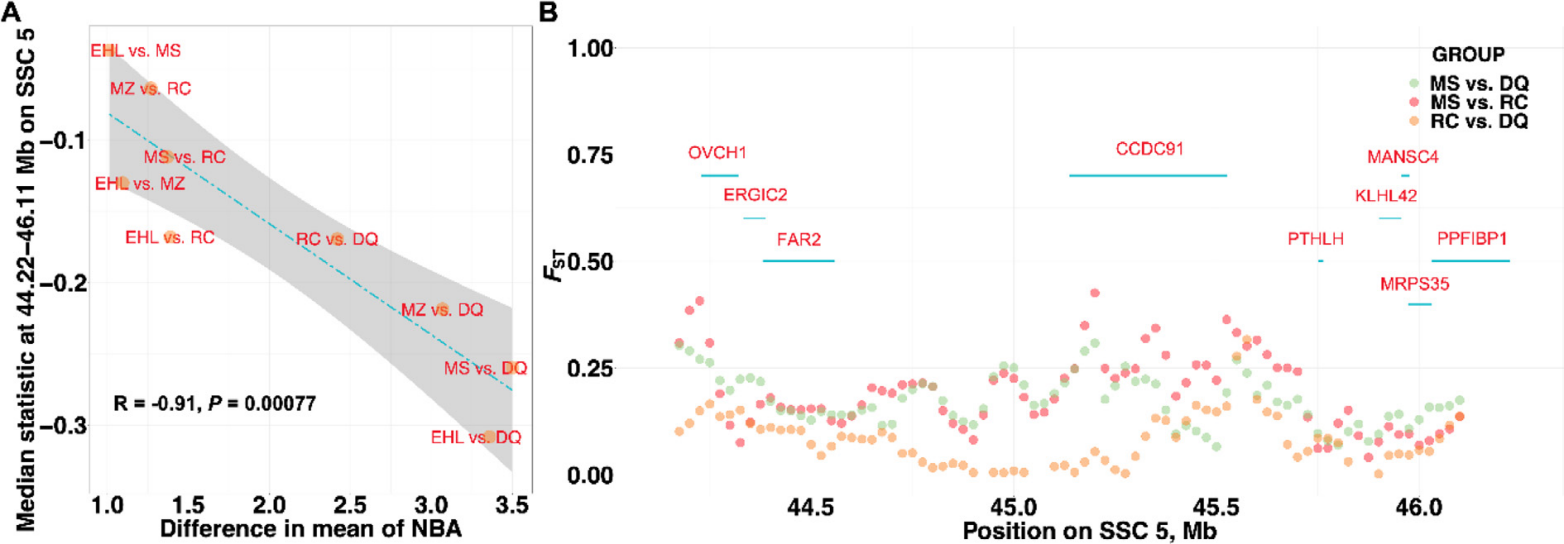
Patterns of selection at the ROHet: 44.22–46.11 Mb on SSC 5. (A) The correlation between the median genetic diversity ratio at the ROHet and the corresponding differences in number born alive trait between each pair of pig breeds; (B) The *F*_ST_ statistics at the ROHet in selected breed comparisons.

We calculated θπ statistic across the highest frequency ROHet in JH, CA, LY, and SX pigs, however, no trend was observed in comparison of these pigs (Supplementary Fig. 5). These might be caused by the similar reproduction performance of these pigs. Thus, we used the high-density genotypes of other pig breeds contained the extremely prolific (MS and EHL), prolific (MZ and RC), and lowest litter sized (DQ) breeds to detect selection signatures. Later, we correlated the median θ_π_ statistic across the highest frequency litter traits-related ROHet for each breed comparison to the difference in mean breed value for TNB, NBA, and TND traits. One ROHet (44.22–46.11 Mb on SSC 5) was significantly associated with TNB and NBA traits (Supplementary Fig. 6). It was also detected in the *F*_ST_ analysis with the breeds of group 1 and contains nine genes: *OVCH1*, *ERGIC2*, *FAR2*, *CCDC91*, *PTHLH*, *KLHL42*, *MANSC4*, *MRPS35*, and *PPFIBP1*. Herein, *OVCH1* and *CCDC91* were two strong candidates with the peak of selection falling (Fig. 4**)**. The similar selection patterns were also shown in the analysis with the breeds of group 2 (Supplementary Fig. 7), precisely revealing the genetic differentiation at the ROHet between the prolific pig breeds and pig breeds with the lowest litter size. Meanwhile, the peak of selection was overlapped with the QTLs that were related to reproduction traits (i.e., teat number, number of stillborn, reproductive tract weight, uterine horn length, and uterine horn weight traits).

### 3.5 Pattern of selection at the highest frequency litter traits-related ROH

**Fig. 5:**
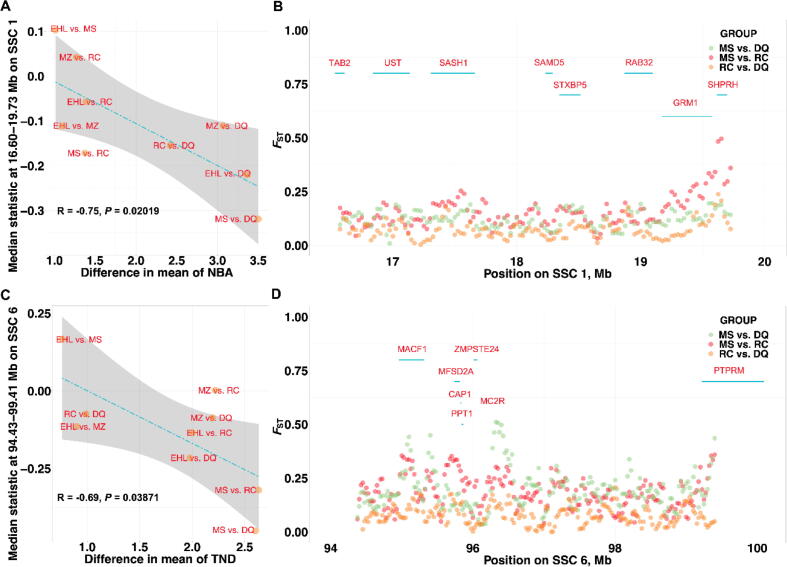
Patterns of selection at the ROH: 16.60–19.73 Mb on SSC 1 and 94.43–99.41 Mb on SSC 6. (A) ‘The correlation between the median genetic diversity ratio at the ROH 16.60–19.73 Mb on SSC 1 and the corresponding differences in number born alive trait between each pair of pig breeds; (B) The *F*_ST_ statistics at the ROH 16.60–19.73 Mb on SSC 1 in selected breed comparisons; (C) The correlation between the median genetic diversity ratio at the ROH 94.43–99.41 Mb on SSC 6 and the corresponding differences in total number of piglets born dead trait between each pair of pig breeds; (D) The *F*_ST_ statistics at the ROH 94.43–99.41 Mb on SSC 6 in selected breed comparisons.

In addition, there was no trend of selection patterns across the highest frequency ROH has been observed in comparison of JH, CA, LY, and SX pigs (Supplementary Fig. 5). Likewise, we investigated the correlation between the median θ_π_ statistic across the highest frequency litter traits-related ROH for each breed and difference in mean breed value for TNB, NBA, and TND traits with other five pig breeds (Supplementary Fig. 8). The median θ_π_ statistic at the ROH: 16.60–19.73 Mb on SSC 1 had a significant correlation with difference in mean of TNB and NBA (Fig. 5A). There were eight promising candidate genes (i.e., *TAB2*, *UST*, *SASH1*, *SAMD5*, *STXBP5*, *RAB32*, *GRM1*, and *SHPRH*) located on the ROH: 16.60–19.73 Mb on SSC 1 (Fig. 5B and Supplementary Fig. 7). The peak of selection across the ROH were overlapped with QTLs of number of stillborn and teat number traits. In the meanwhile, we observed the significant correlation between the median θ_π_ statistic for each breed (Fig. 5C) and TND at the ROH: 94.43–99.41 Mb on SSC 6 where 43 promising candidate genes (e.g., *MACF1*, *MFSD2A*, *CAP1*, *PPT1*, *ZMPSTE24*, *MC2R*, and *PTPRM*) and a set of QTLs of reproduction traits (i.e., age at puberty, gestation length, litter size, non-functional nipples, number of mummified pigs, and teat number traits) were located (Fig. 5D). The further detection of selection signatures with group 2 proved the accuracy of the genetic differentiation between the extremely, prolific pig breeds, and pig breeds with the lowest litter size detected by *F*_ST_ analysis with group 1 (Supplementary Fig. 7).

### 3.6 The potential roles of the promising candidate genes in reproductive function

We further explored the potential roles of the genes (i.e., *CCDC91*, *SASH1*, *SAMD5*, *MACF1*, *MFSD2A*, *EPC2*, and *MBD5*) that have been recorded in the webTWAS database and not been widely reported to be fertility-related genes (Table 2). Interestingly, we found that the promising candidate genes were associated with polycystic ovary syndrome, thyroid problem, and diabetes.

**Table 2:** The potential roles of the genes that have not been widely reported to be fertilize-related genes.

Gene symbol	Pig gene ID	Human Gene ID	Associated disease	Associated tissues
*CCDC91*	ENSSSCG00000024232	ENSG00000123106	Polycystic ovary syndrome [52], Type 2 Diabetes [53], Osteoarthritis [54]	Ovary, testis, brain hippocampus
*SASH1*	ENSSSCG00000033175	ENSG00000123106	Thyroid problem, Hypothyroidism/myxoedema [55]	Thyroid, brain hippocampus
*SAMD5*	ENSSSCG00000004112	ENSG00000203727	Thyroid problem, Local infections of skin and subcutaneous tissue, Diseases of veins, lymphatic vessels and lymph nodes [55]	Brain hypothalamus, spleen, testis
*MACF1*	ENSSSCG00000003654	ENSG00000127603	Type 2 Diabetes, Diabetes [56], hypertensive diseases [55]	Ovary, pancreas, thyroid, brain hippocampus, pituitary, spleen, liver
*MFSD2A*	ENSSSCG00000003669	ENSG00000168389	Type 2 Diabetes [53], Diabetes mellitus, hypertension [55], diabetes [56]	Uterus
*EPC2*	ENSSSCG00000027211	ENSG00000135999	Diabetes [56]	Liver
*MBD5*	ENSSSCG00000015667	ENSG00000204406	Diabetes mellitus [55]	Colon Sigmoid

**Fig. 6:**
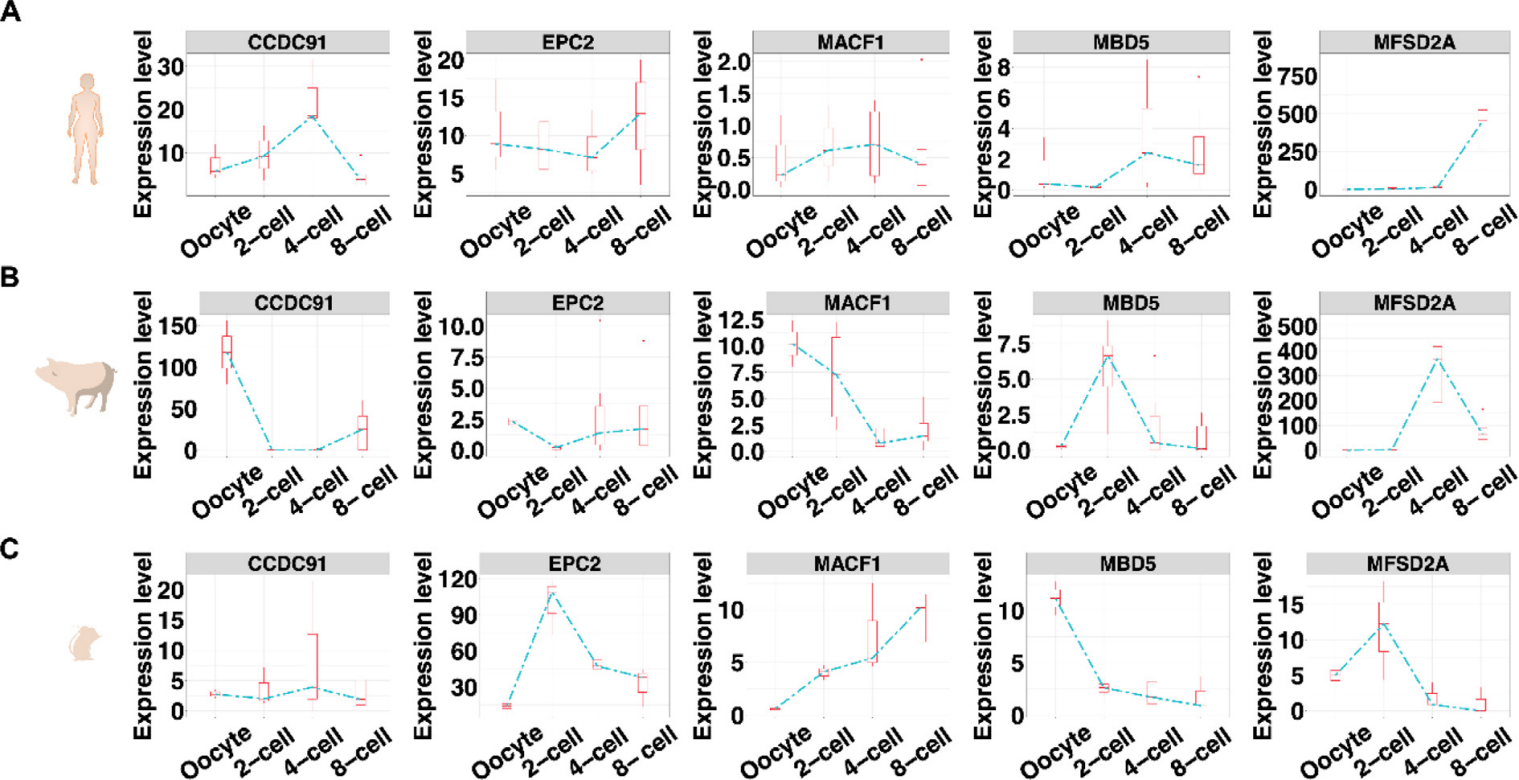
Comparative transcriptome analysis on the promising candidate genes. The expression level of *CCDC91*, *MACF1*, *MFSD2A*, *EPC2*, and *MBD5* during the development of early embryo in human (A), pig (B), and mouse (C).

According to the associated diseases and tissues given in the database, we further explored the changes in expression level of the genes from oocyte to 8-cell in three mammalian species (i.e., human, pig, and mouse), and the differences in expression level of the genes between GDM and healthy control placentas. We observed that the expression level of *CCDC91*, *MACF1*, *MFSD2A*, *EPC2*, and *MBD5* has a significant fluctuation during the development of early embryo in at least one out of three species (Fig. 6A-C). In addition, *SASH1* and *SAMD5* were only detected from oocyte to 8-cell in mouse and showed a significantly high expression level at the period of oocyte comparison with other periods (Supplementary Fig. 9). The results elucidated the potential roles of the promising candidate genes in the early embryonic development of mammals. Furthermore, the expression level of *MBD5* and *SAMD5* were significantly different (*t*-test, *P*-value = 0.03549 and 0.01884, respectively) between GDM and healthy control placentas (Supplementary Fig. 10).

## 4 Discussion

### 4.1 The factors affecting ROHet and ROH detection

In general, genotyping errors, the minimal SNP density per segment, the minimum length of a ROHet or ROH, the number of genotyping errors allowed and the minimum number of SNPs included in a ROHet or ROH are likely to affect the detection of ROHet and ROH [17], [57], [58], [59]. These factors are especially relevant for populations with fewer number of individuals, which may lack pedigree records, therefore, genomic analyses help fill the gap to their genetic characterization. The density of the SNP chip is related to the minimum ROH length, fewer and shorter ROH will be detected with higher density of SNP [57]. Furthermore, the minimal SNP density affect genome coverage of the ROH analysis and is crucial for medium density genotypes [33]. With medium density SNP panel, one heterozygous call is allowed since genotyping errors in SNP chip data may occur [57], and allowing a number of genotyping errors in long ROH may minimize the underestimation of them [58]. In addition, Meyermans et al. [33] has shown that MAF and LD pruning severely impact ROH analyses and recommend to avoid MAF and LD pruning prior to ROH analyses based on medium density genotypes. Meanwhile, ROHet has far less characterized than ROH in livestock, especially in pigs [16], [60]. Biscarini et al. [57] presented the results of the first sensitivity analysis for ROHet and varied the number of missing and homozygous SNPs allowed inside ROHet. In comparison with the detection of ROH, they found a significant increase in the number and average size of the detected ROHet when increasing numbers of missing and/or homozygous SNPs are allowed.

### 4.2 Distribution of heterozygous and homozygous loci

According to previous studies [61], [62], indigenous pigs studied in this study had lower heterozygosity than intensively-reared commercial pig breeds. The results of ROHet detection showed that a few ROHet were detected in indigenous pigs, except LY pigs, which might have gene introgression from intensively-reared commercial pig breeds (data not shown). A study reported that Mangalarga Marchador horses were inbred, leading to reduced heterozygosity and only a few ROHet were formed [19]. In this study, the number of ROHet were lower than that of ROH, which reflected the breed conservation of the studied pig populations. In addition, the number and length of ROH or ROHet had negative correlations, which have been observed in previous studies [57], [63]. In spite of this heterogeneity of methodology and data, all studies found that heterozygosity-regions were much rarer and shorter compared to ROH.

### 4.3 The potential roles of the shared ROHet and ROH

The shared ROHet showed the possibility of similar selection patterns on growth, meat quality, health, and reproduction traits in Chinese indigenous pigs. Here, we noticed the tissue-specific genes located on the shared ROHet, which might reflect the same breed characteristics of indigenous pigs more typically. The *ceruloplasmin* (*CP*) gene potential participated in iron transport during spleen development [64] and the growth of piglets [65]. The *timeless circadian regulator* (*TIMELESS*) gene was linked to cell survival after damage or stress [66], which might help disease resistance of indigenous pigs. Two testis tissue-specific genes, the *testis specific serine kinase substrate* (*TSKS*) and *dynein axonemal heavy chain 17* (*DNAH17*) genes, were essential for spermatogenesis and fertility in mammals [67], [68].

Likewise, we investigated the tissue-specific genes at the shared ROH to uncover the same breed characteristics of the studied populations. The *ubiquitin like with PHD and ring finger domains 1* (*UHRF1*) gene involved in porcine adipogenesis [69]. The *GC vitamin D binding protein* (*GC*) gene was associated with bone metabolic pathways [70]. The *alpha fetoprotein* (*AFP*) gene encodes a major fetal protein and may promote fetal ovarian follicular development in Chinese indigenous pigs [71]. To sum up, the shared ROH might cause broadly similar fat deposition, litter size and body length characteristics in indigenous pigs.

### 4.4 Litter traits-related ROHet and ROH detection

In this study, we highlighted the genetic characteristics of litter traits in indigenous pigs. The highest frequency litter traits-related ROHet within the genome might be able to achieve optimal production performance in indigenous pigs. We identified three highest frequency litter traits-related ROHet among parity groups and each litter traits. Several promising candidate genes at the ROHet involved in reproductive pathways in mammals. The *calcium/calmodulin dependent protein kinase II delta* (*CAMK2D*) gene participated in GnRH signalling pathway, as one of the most important reproductive pathways [72]. The *UDP glycosyltransferase 8* (*UGT8*) gene was down-regulated in the endometrium of women affected by implantation failure [73]. Whereas, we noticed that these genes had pleiotropic effects on the growth of muscle and fat deposition in adipose tissues [74], [75]. This implied these genes might lead to lighter piglets born, and give birth to more piglets.

High levels of inbreeding are directly related to a higher incidence of ROH, which could result in inbreeding depression [76]. Previous studies reported several genes likely caused fetal mortality in mutant homozygous pigs [77]. Here, the roles of the *activin A receptor type 2A* (*ACVR2A*), *kinesin family member 5C* (*KIF5C*) and *Metabolism of cobalamin associated D* (*MMADHC*) gene in reproductive pathways have been previously reported [78], [79], [80]. In the meantime, we reported for the first time the potential effects of other two promising candidate genes (*EPC2* and *MBD5*) on fertility.

In addition, the analyses of association degree between the highest frequency ROHet (or ROH) and litter traits revealed that the genomic structures of piglets born alive and dead were different among parity groups, which were consistent with previous studies [3], [81]. In view of the vary genetic background of traits of born alive and dead among parities, the different parities should be considered as different traits to proceed selection and breeding works.

### 4.5 Porcine litter traits-related regions detection via selection signatures

Furthermore, we deeply investigated the roles of the highest frequency litter traits-related ROHet and ROH in pigs via litter traits-specific selection signatures. There was no trend of selection patterns has been observed in comparison of the θπ of JH, CA, LY, and SX pigs across the highest frequency ROHet and ROH. These might be caused by the similar reproduction performance of these pigs. Thus, we used the high-density genotypes of other five pig breeds with different levels of reproduction performance to detect selection signatures. A set of known and novel candidate genes associated with litter size were located on the peak of selection. The *ovochymase 1* (*OVCH1*) and *coiled-coil domain containing 91* (*CCDC91*) genes were two strong candidates with the peak of selection across the ROHet that were related to TNB and NBA traits. Herein, The *OVCH1* gene encodes oocyte extracellular polyproteins and has pleiotropic effects on fertility and the development of muscle [82], [83]. And, it has been established that the *CCDC91* gene involved in skeletal development [84], but its potential role in litter traits remains unknown.

As mentioned above, the genes of which were homozygous might lead to fetal mortality. The ROH: 16.60–19.73 Mb on SSC 1 and ROH: 94.43–99.41 Mb on SSC 6 were overlapped with several QTLs of the loss of reproduction traits, e.g., non-functional nipples, number of mummified pigs, and number of stillborn. In addition, the promising candidate genes located across the ROH were associated with embryonic and fetal development. The *microtubule actin crosslinking factor 1* (*MACF1*) gene has been linked to ovarian function [85]. The *major facilitator superfamily domain containing 2a* (*MFSD2A*) gene was needed in body growth, lipid metabolism and brain integrity [86]. The TGF-beta activated kinase 1 binding protein 2 (TAB2) gene were associated with non-heterotaxic foetuses [87].

### 4.6 The promising candidate genes with reproductive function

Previous studies have showed that the homozygous variants could lead to human infertility with early embryonic arrest [88] and zygotic cleavage failure [89]. In this study, we deeply explored the potential fertility-related function of the promising candidate genes that have not been widely investigated. The expression level of *CCDC91*, *MACF1*, *MFSD2A*, *EPC2*, and *MBD5* has a significant fluctuation during the development of early embryo, but showed different trends in human, pig, and mouse. The phenomenon was consistent with previous studies, which stated that the molecular mechanisms of embryonic development shared many similarities, but also several marked differences in mammals [90], [91]. In addition, the infants born to GDM females had reduced size at birth and persisting reductions in adiposity [92]. *MBD5* and *SAMD5* likely involved in the occurrence of GDM. The results showed that the promising candidate genes played potential roles in mammalian fertility-related pathways.

### 4.7 The use of the promising biological priors

To our knowledge, few ROHet and ROH analyses have been systematically conducted in pigs. And amounts of “omics” data were generated and released by some consortiums (e.g., the FarmGTEx [93] and FAANG consortium [94]), providing opportunities to explore the potential roles of the causal genome regions and promising candidate genes. Here, we integrated the findings of ROHet and ROH with transcriptome analyses to better elucidate the potential causal genes that determine the genetic and phenotypic differences among animals. Many studies have proved that modifying the models to incorporate prior knowledge (e.g., the potential causal genes) improves the performance of genomic prediction or selection [95], [96], [97]. Meanwhile, animal models have been widely used for the understanding of human physiological processes. The porcine anatomy and physiology are similar to human anatomy and physiology (e.g., all-eating and hormonal cycle), therefore, some breeds with size and weight similar to human adults making the breeds attractive in biomedical research [98], [99]. In this study, the in-depth cross-species transcriptome comparison reveals the conserved and divergent features of the promising candidate genes in human, mouse, and pig, the results will benefit animal model selection for fertility-related traits and/or diseases.

Taken together, we present a multi-omics analyses integrating transcriptome and genome analyses that captures the underlying chromosome regions of litter traits. The in-depth analyses provided the evidence for the roles of the promising candidate genes that can be used as biological priors to improve the accuracies of genomic selection and further benefit pig genetic improvement. Besides, the results offer a genome-wide understanding of the relationships between heterozygosity or homozygosity regions and reproductive success and the loss of reproduction in mammals.

## Declaration of Competing Interest

The authors declare that they have no known competing financial interests or personal relationships that could have appeared to influence the work reported in this paper.

## References

[1] Chen Z., Li Y., Zhang Z., Zhao W., Zhang Z., Xiang Y. (2021). Genome-wide epistatic interactions of litter size at birth in Chinese indigenous pigs. Anim Genet.

[2] Zheng X., Zhao P., Yang K., Ning C., Wang H., Zhou L. (2020). CNV analysis of Meishan pig by next-generation sequencing and effects of AHR gene CNV on pig reproductive traits. J Anim Sci Biotechnol.

[3] Chen Z., Ye S., Teng J., Diao S., Yuan X., Chen Z. (2019). Genome-wide association studies for the number of animals born alive and dead in duroc pigs. Theriogenology.

[4] Norseeda W., Liu G., Teltathum T., Supakankul P., Sringarm K., Naraballobh W. (2021). Association of il-4 and il-4r polymorphisms with litter size traits in pigs. Animals.

[5] Ding R., Qiu Y., Zhuang Z., Ruan D., Wu J., Zhou S. (2021). Genome-wide association studies reveals polygenic genetic architecture of litter traits in Duroc pigs. Theriogenology.

[6] Wu P., Wang K., Zhou J., Yang Q., Yang X., Jiang A. (2019). A genome wide association study for the number of animals born dead in domestic pigs. BMC Genet.

[7] Sell-Kubiak E., Duijvesteijn N., Lopes M.S., Janss L.L.G., Knol E.F., Bijma P. (2015). Genome-wide association study reveals novel loci for litter size and its variability in a Large White pig population. BMC Genomics.

[8] Ye J., Tan C., Hu X., Wang A., Wu Z. (2018). Genetic parameters for reproductive traits at different parities in Large White Pigs. J Anim Sci.

[9] Sell-Kubiak E. (2021). Selection for litter size and litter birthweight in Large White pigs: Maximum, mean and variability of reproduction traits. Animal.

[10] Zhang C., Ye H.e., Huang Y. (2019). Genome-wide association study for reproductive traits in a duroc pig population. Animals.

[11] Zhang Z., Ober U., Erbe M., Zhang H., Gao N., He J. (2014). Improving the accuracy of whole genome prediction for complex traits using the results of genome wide association studies. PLoS ONE.

[12] Amaral A.F., McQueen B.E., Bellingham-Johnstun K., Poston T.B., Darville T., Nagarajan U.M. (2021). Host-pathogen interactions of chlamydia trachomatis in porcine oviduct epithelial cells. Pathogens.

[13] Zhou X., He Y., Li N., Bai G., Pan X., Zhang Z. (2021). DNA methylation mediated RSPO2 to promote follicular development in mammals. Cell Death Dis.

[14] Samuels D.C., Wang J., Ye F., He J., Levinson R.T., Sheng Q. (2016). Heterozygosity ratio, a robust global genomic measure of autozygosity and its association with height and disease risk. Genetics.

[15] Marras G., Wood B.J., Makanjuola B., Malchiodi F., Peeters K., Van A.P. (2018). 11th World Congr Genet Appl to Livest Prod.

[16] Sanglard L.P., Huang Y., Gray K.A., Linhares D.C.L., Dekkers J.C.M., Niederwerder M.C. (2021). Further host-genomic characterization of total antibody response to PRRSV vaccination and its relationship with reproductive performance in commercial sows: genome-wide haplotype and zygosity analyses. Genet Sel Evol.

[17] Peripolli E., Munari D.P., Silva M.V.G.B., Lima A.L.F., Irgang R., Baldi F. (2017). Runs of homozygosity: current knowledge and applications in livestock. Anim Genet.

[18] Forutan M., Ansari Mahyari S., Baes C., Melzer N., Schenkel F.S., Sargolzaei M. (2018). Inbreeding and runs of homozygosity before and after genomic selection in North American Holstein cattle. BMC Genomics.

[19] Bizarria dos Santos W., Pimenta Schettini G., Fonseca M.G., Pereira G.L., Loyola Chardulo L.A., Rodrigues Machado Neto O. (2021). Fine-scale estimation of inbreeding rates, runs of homozygosity and genome-wide heterozygosity levels in the Mangalarga Marchador horse breed. J Anim Breed Genet.

[20] Nosrati M., Asadollahpour Nanaei H., Javanmard A., Esmailizadeh A. (2021). The pattern of runs of homozygosity and genomic inbreeding in world-wide sheep populations. Genomics.

[21] Yang H.C., Chang L.C., Liang Y.J., Lin C.H., Wang P.L. (2012). A genome-wide homozygosity association study identifies runs of homozygosity associated with rheumatoid arthritis in the human major histocompatibility complex. PLoS ONE.

[22] Wen J., Zheng Z., Gong M., Li D., Hu S., Cai Y. (2022). Ancient genomes reveal the genetic inheritance and recent introgression in Chinese indigenous pigs. Sci China Life Sci.

[23] Ai H., Fang X., Yang B., Huang Z., Chen H., Mao L. (2015). Adaptation and possible ancient interspecies introgression in pigs identified by whole-genome sequencing. Nat Genet.

[24] Jiang Y., Li X., Liu J., Zhang W., Zhou M., Wang J. (2022). Genome-wide detection of genetic structure and runs of homozygosity analysis in Anhui indigenous and Western commercial pig breeds using PorcineSNP80k data. BMC Genomics.

[25] Wu W., Zhan J., Tang X., Li T., Duan S. (2022). Characterization and identification of pork flavor compounds and their precursors in Chinese indigenous pig breeds by volatile profiling and multivariate analysis. Food Chem.

[26] Liu C., Li P., Zhou W., Ma X., Wang X., Xu Y. (2020). Genome data uncover conservation status, historical relatedness and candidate genes under selection in chinese indigenous pigs in the taihu lake region. Front Genet.

[27] Huang M., Zhang H., Wu Z.P., Wang X.P., Li D.S., Liu S.J. (2021). Whole-genome resequencing reveals genetic structure and introgression in Pudong White pigs. Animal.

[28] Zhao P., Yu Y., Feng W., Du H., Yu J., Kang H. (2018). Evidence of evolutionary history and selective sweeps in the genome of Meishan pig reveals its genetic and phenotypic characterization. GigaScience.

[29] Chen H., Huang M., Yang B., Wu Z., Deng Z., Hou Y. (2020). Introgression of Eastern Chinese and Southern Chinese haplotypes contributes to the improvement of fertility and immunity in European modern pigs. GigaScience.

[30] Dobrzański J., Mulder H.A., Knol E.F., Szwaczkowski T., Sell-Kubiak E. (2020). Estimation of litter size variability phenotypes in Large White sows. J Anim Breed Genet.

[31] VanRaden P.M. (2008). Efficient methods to compute genomic predictions. J Dairy Sci.

[32] Purcell S., Neale B., Todd-Brown K., Thomas L., Ferreira M.A.R., Bender D. (2007). PLINK: a tool set for whole-genome association and population-based linkage analyses. Am J Hum Genet.

[33] Meyermans R., Gorssen W., Buys N., Janssens S. (2020). How to study runs of homozygosity using plink? a guide for analyzing medium density snp data in livestock and pet species. BMC Genom..

[34] Li H., Durbin R. (2010). Fast and accurate long-read alignment with Burrows-Wheeler transform. Bioinformatics.

[35] Li H. (2011). A statistical framework for SNP calling, mutation discovery, association mapping and population genetical parameter estimation from sequencing data. Bioinformatics.

[36] McKenna A., Hanna M., Banks E., Sivachenko A., Cibulskis K., Kernytsky A. (2010). The genome analysis toolkit: a mapreduce framework for analyzing next-generation DNA sequencing data. Genome Res.

[37] Marras G., Gaspa G., Sorbolini S., Dimauro C., Ajmone-Marsan P., Valentini A. (2015). Analysis of runs of homozygosity and their relationship with inbreeding in five cattle breeds farmed in Italy. Anim Genet.

[38] Lencz T., Lambert C., DeRosse P., Burdick K.E., Morgan T.V., Kane J.M. (2007). Runs of homozygosity reveal highly penetrant recessive loci in schizophrenia. Proc Natl Acad Sci U S A.

[39] Dumitrescu A.M., Liao X.H., Abdullah M.S.Y., Lado-Abeal J., Majed F.A., Moeller L.C. (2005). Mutations in SECISBP2 result in abnormal thyroid hormone metabolism. Nat Genet.

[40] Storey J.D., Xiao W., Leek J.T., Tompkins R.G., Davis R.W. (2005). Significance analysis of time course microarray experiments. Proc Natl Acad Sci U S A.

[41] International Encyclopedia of Statistical Science. 2011. https://doi.org/10.1007/978-3-642-04898-2.

[42] Team RC (2021). R: A Language and Environment for Statistical Computing. R Found Stat Comput.

[43] Danecek P., Auton A., Abecasis G., Albers C.A., Banks E., DePristo M.A. (2011). The variant call format and VCFtools. Bioinformatics.

[44] Weir B.S., Cockerham C.C. (1984). Estimating F-statistics for the analysis of population structure. Evolution (N Y).

[45] Karlsson M., Sjöstedt E., Oksvold P., Sivertsson Å., Huang J., Álvez M.B. (2022). Genome-wide annotation of protein-coding genes in pig. BMC Biol.

[46] Cao C., Wang J., Kwok D., Cui F., Zhang Z., Zhao D. (2022). WebTWAS: A resource for disease candidate susceptibility genes identified by transcriptome-wide association study. Nucleic Acids Res.

[47] Xue Z., Huang K., Cai C., Cai L., Jiang C.Y., Feng Y. (2013). Genetic programs in human and mouse early embryos revealed by single-cell RNA sequencing. Nature.

[48] Kong Q., Yang X., Zhang H., Liu S., Zhao J., Zhang J. (2020). Lineage specification and pluripotency revealed by transcriptome analysis from oocyte to blastocyst in pig. FASEB J.

[49] Kim D., Langmead B., Salzberg S.L. (2015). HISAT: a fast spliced aligner with low memory requirements. Nat Methods.

[50] Liao Y., Smyth G.K., Shi W. (2014). FeatureCounts: An efficient general purpose program for assigning sequence reads to genomic features. Bioinformatics.

[51] Yu G., Wang L.-G., Han Y., He Q.-Y. (2012). clusterProfiler: an R Package for Comparing Biological Themes Among Gene Clusters. Omi A J Integr Biol.

[52] Day F., Karaderi T., Jones M.R., Meun C., He C., Drong A. (2018). Large-scale genome-wide meta-analysis of polycystic ovary syndrome suggests shared genetic architecture for different diagnosis criteria. PLoS Genet.

[53] Xue A., Wu Y., Zhu Z., Zhang F., Kemper K.E., Zheng Z. (2018). Genome-wide association analyses identify 143 risk variants and putative regulatory mechanisms for type 2 diabetes. Nat Commun.

[54] Tachmazidou I., Hatzikotoulas K., Southam L., Esparza-Gordillo J., Haberland V., Zheng J. (2019). Identification of new therapeutic targets for osteoarthritis through genome-wide analyses of UK Biobank data. Nat Genet.

[55] Canela-Xandri O., Rawlik K., Tenesa A. (2018). An atlas of genetic associations in UK Biobank. Nat Genet.

[56] Watanabe K., Stringer S., Frei O., Umićević Mirkov M., de Leeuw C., Polderman T.J.C. (2019). A global overview of pleiotropy and genetic architecture in complex traits. Nat Genet.

[57] Biscarini F., Mastrangelo S., Catillo G., Senczuk G., Ciampolini R. (2020). Insights into genetic diversity, runs of homozygosity and heterozygosity-rich regions in maremmana semi-feral cattle using pedigree and genomic data. Animals.

[58] Ferenčaković M., Sölkner J., Curik I. (2013). Estimating autozygosity from high-throughput information: effects of SNP density and genotyping errors. Genet Sel Evol.

[59] Zhang Z., Zhang Q., Xiao Q., Sun H., Gao H., Yang Y. (2018). Distribution of runs of homozygosity in Chinese and Western pig breeds evaluated by reduced-representation sequencing data. Anim Genet.

[60] Ruan D., Yang J., Zhuang Z., Ding R., Huang J., Quan J. (2021). Assessment of heterozygosity and genome-wide analysis of heterozygosity regions in two duroc pig populations. Front Genet.

[61] Chen J., Peng J., Xiao Q., Pan Y., Zhang X., Lo L.J. (2018). The genetic diversity and population structures of indigenous pig breeds in Zhejiang Province revealed by GGRS sequencing. Anim Genet.

[62] Zhang Z., Xiao Q., Zhang Q., Sun H., Chen J., Li Z. (2018). Genomic analysis reveals genes affecting distinct phenotypes among different Chinese and western pig breeds. Sci Rep.

[63] Huo J.L., Zhang L.Q., Zhang X., Wu X.W., Ye X.H., Sun Y.H. (2022). Genome-wide single nucleotide polymorphism array and whole-genome sequencing reveal the inbreeding progression of Banna minipig inbred line. Anim Genet.

[64] Che T., Li D., Jin L., Fu Y., Liu Y., Liu P. (2018). Long non-coding RNAs and mRNAs profiling during spleen development in pig. PLoS ONE.

[65] Tao X., Xu Z., Men X. (2015). Transient changes of enzyme activities and expression of stress proteins in the small intestine of piglets after weaning. Arch Anim Nutr.

[66] Fabbri M.C., Dadousis C., Tiezzi F., Maltecca C., Lozada-Soto E., Biffani S. (2021). Genetic diversity and population history of eight Italian beef cattle breeds using measures of autozygosity. PLoS ONE.

[67] Chen L., Ouyang J., Li X., Xiao X., Sun W., Li S. (2021). DNAH17 is essential for rat spermatogenesis and fertility. J Genet.

[68] Hao Z., Jha K.N., Kim Y.H., Vemuganti S., Westbrook V.A., Chertihin O. (2004). Expression analysis of the human testis-specific serine/threonine kinase (TSSK) homologues. A TSSK member is present in the equatorial segment of human sperm. Mol Hum Reprod.

[69] Stachecka J., Lemanska W., Noak M., Szczerbal I. (2020). Expression of key genes involved in DNA methylation during in vitro differentiation of porcine mesenchymal stem cells (MSCs) into adipocytes. Biochem Biophys Res Commun.

[70] Delanghe J.R., Speeckaert R., Speeckaert M.M. (2015). Behind the scenes of vitamin D binding protein: more than vitamin D binding. Best Pract Res Clin Endocrinol Metab.

[71] Xu M., Che L., Wang D., Yang Z., Zhang P., Lin Y. (2015). Proteomic analysis of fetal ovary reveals that ovarian developmental potential is greater in Meishan pigs than in Yorkshire pigs. PLoS ONE.

[72] Tahir M.S., Porto-Neto L.R., Gondro C., Shittu O.B., Wockner K., Tan A.W.L. (2021). Meta-analysis of heifer traits identified reproductive pathways in bos indicus cattle. Genes (Basel).

[73] Maekawa R., Taketani T., Mihara Y., Sato S., Okada M., Tamura I. (2017). Thin endometrium transcriptome analysis reveals a potential mechanism of implantation failure. Reprod Med Biol.

[74] Lee Y.S., Shin D., Song K.D. (2018). Dominance effects of ion transport and ion transport regulator genes on the final weight and backfat thickness of Landrace pigs by dominance deviation analysis. Genes Genom..

[75] Arora D., Srikanth K., Lee J., Lee D., Park N., Wy S. (2021). Integration of multi-omics approaches for functional characterization of muscle related selective sweep genes in Nanchukmacdon. Sci Rep.

[76] Curik I., Ferenčaković M., Sölkner J. (2014). Inbreeding and runs of homozygosity: a possible solution to an old problem. Livest Sci.

[77] Derks M.F.L., Lopes M.S., Bosse M., Madsen O., Dibbits B., Harlizius B. (2018). Balancing selection on a recessive lethal deletion with pleiotropic effects on two neighboring genes in the porcine genome. PLoS Genet.

[78] Li X., Ye J., Han X., Qiao R., Li X., Lv G. (2020). Whole-genome sequencing identifies potential candidate genes for reproductive traits in pigs. Genomics.

[79] Kant K., Tomar A.K., Sharma P., Kundu B., Singh S., Yadav S. (2019). Human epididymis protein 4 quantification and interaction network analysis in seminal plasma. Protein Pept Lett.

[80] Pupavac M., Garcia M.A.M., Rosenblatt D.S., Jerome-Majewska L.A. (2011). Expression of mmachc and mmadhc during mouse organogenesis. Mol Genet Metab.

[81] Roehe R., Kennedy B.W. (1995). Estimation of genetic parameters for litter size in Canadian Yorkshire and Landrace swine with each parity of farrowing treated as a different trait. J Anim Sci.

[82] Varade J., Wang N., Lim C.K., Zhang T., Zhang Y., Liu X. (2018). Novel genetic loci associated HLA-B*08:01 positive myasthenia gravis. J Autoimmun.

[83] Mino M., Sawada H. (2016). Follicle cell trypsin-like protease HrOvochymase: its cDNA cloning, localization, and involvement in the late stage of oogenesis in the ascidian Halocynthia roretzi. Mol Reprod Dev.

[84] Hsu Y.H., Estrada K., Evangelou E., Ackert-Bicknell C., Akesson K., Beck T. (2019). Meta-analysis of genomewide association studies reveals genetic variants for hip bone geometry. J Bone Miner Res.

[85] Jaillard S., Bell K., Akloul L., Walton K., McElreavy K., Stocker W.A. (2020). New insights into the genetic basis of premature ovarian insufficiency: novel causative variants and candidate genes revealed by genomic sequencing. Maturitas.

[86] Gázquez A., Ruíz-Palacios M., Larqué E. (2017). DHA supplementation during pregnancy as phospholipids or TAG produces different placental uptake but similar fetal brain accretion in neonatal piglets. Br J Nutr.

[87] Liu H., Giguet-Valard A.G., Simonet T., Szenker-Ravi E., Lambert L., Vincent-Delorme C. (2020). Next-generation sequencing in a series of 80 fetuses with complex cardiac malformations and/or heterotaxy. Hum Mutat.

[88] Xu Y., Wang R., Pang Z., Wei Z., Sun L., Li S. (2022). Novel homozygous PADI6 variants in infertile females with early embryonic arrest. Front Cell Dev Biol.

[89] Zheng W., Zhou Z., Sha Q., Niu X., Sun X., Shi J. (2020). Homozygous mutations in BTG4 cause zygotic cleavage failure and female infertility. Am J Hum Genet.

[90] Oestrup O., Hall V., Petkov S.G., Wolf X.A., Hyldig S., Hyttel P. (2009). From zygote to implantation: morphological and molecular dynamics during embryo development in the pig. Reprod Domest Anim.

[91] Carreiro L.E., Dos S.GS., Luedke F.E., Goissis M.D. (2021). Cell differentiation events in pre-implantation mouse and bovine embryos. Anim Reprod.

[92] Prentice P.M., Olga L., Petry C.J., Simmons D., Murphy H.R., Hughes I.A. (2019). Reduced size at birth and persisting reductions in adiposity in recent, compared with earlier, cohorts of infants born to mothers with gestational diabetes mellitus. Diabetologia.

[93] Liu S., Gao Y., Canela-Xandri O., Wang S., Yu Y., Cai W. (2020). A comprehensive catalogue of regulatory variants in the cattle transcriptome. BioRxiv.

[94] Clark E.L., Archibald A.L., Daetwyler H.D., Groenen M.A.M., Harrison P.W., Houston R.D. (2020). From FAANG to fork: application of highly annotated genomes to improve farmed animal production. Genome Biol.

[95] Teng J., Huang S., Chen Z., Gao N., Ye S., Diao S. (2020). Optimizing genomic prediction model given causal genes in a dairy cattle population. J Dairy Sci.

[96] Fang L., Sahana G., Ma P., Su G., Yu Y., Zhang S. (2017). Use of biological priors enhances understanding of genetic architecture and genomic prediction of complex traits within and between dairy cattle breeds. BMC Genomics.

[97] Teng J., Ye S., Gao N., Chen Z., Diao S., Li X. (2022). Incorporating genomic annotation into single-step genomic prediction with imputed whole-genome sequence data. J Integr Agric.

[98] Bertho N., Meurens F. (2021). The pig as a medical model for acquired respiratory diseases and dysfunctions: an immunological perspective. Mol Immunol.

[99] Gabriel G.C., Devine W., Redel B.K., Whitworth K.M., Samuel M., Spate L.D. (2021). Cardiovascular development and congenital heart disease modeling in the pig. J Am Heart Assoc.

