# Supplementary figures and images for: Heterozygosity and homozygosity regions affect reproductive success and the loss of reproduction: A case study with litter traits in pigs
Source: Comput Struct Biotechnol J. 2022 Jul 26;20:4060–71. doi: 10.1016/j.csbj.2022.07.039 (PMC9364102; doi:10.1016/j.csbj.2022.07.039)

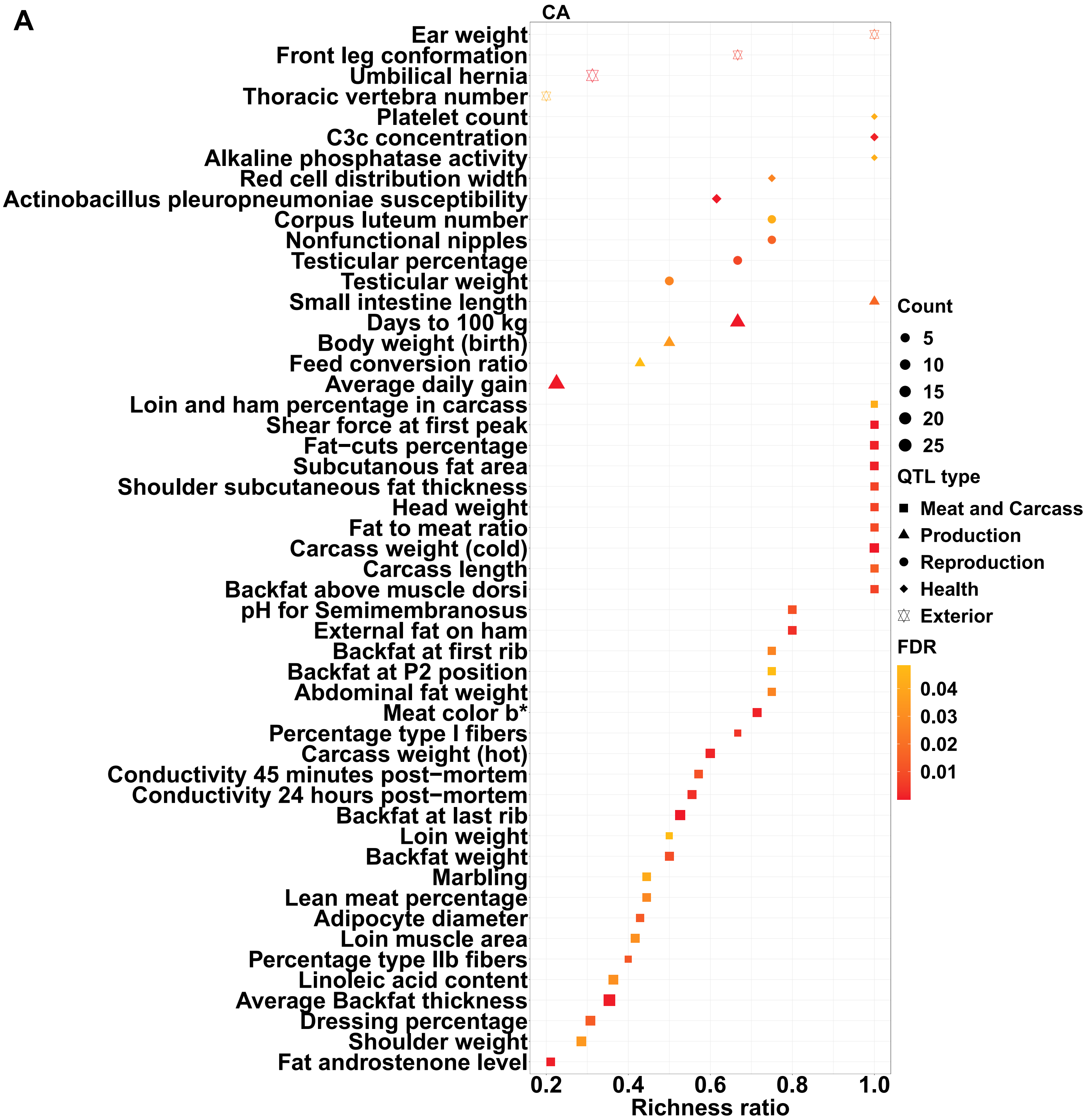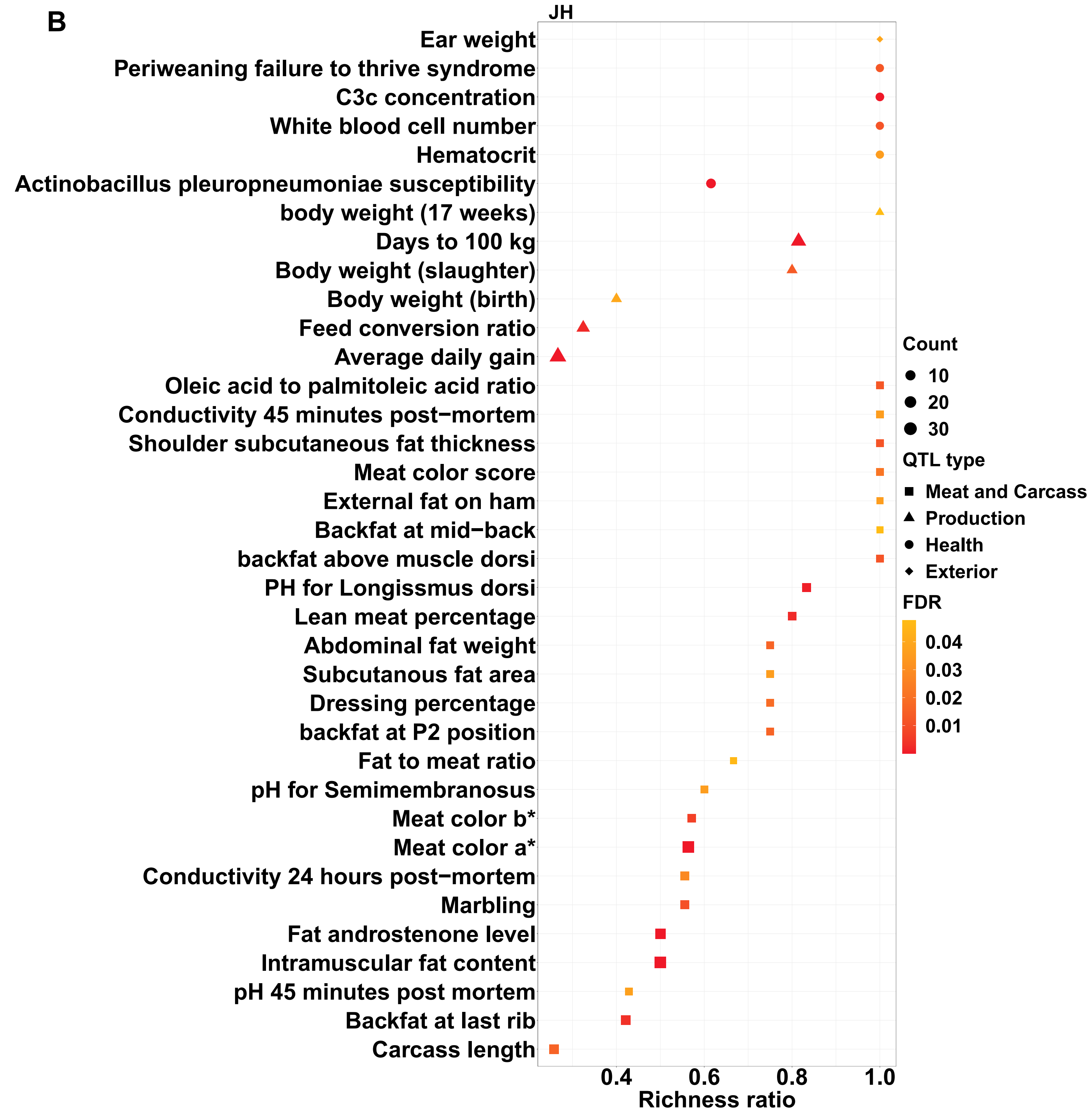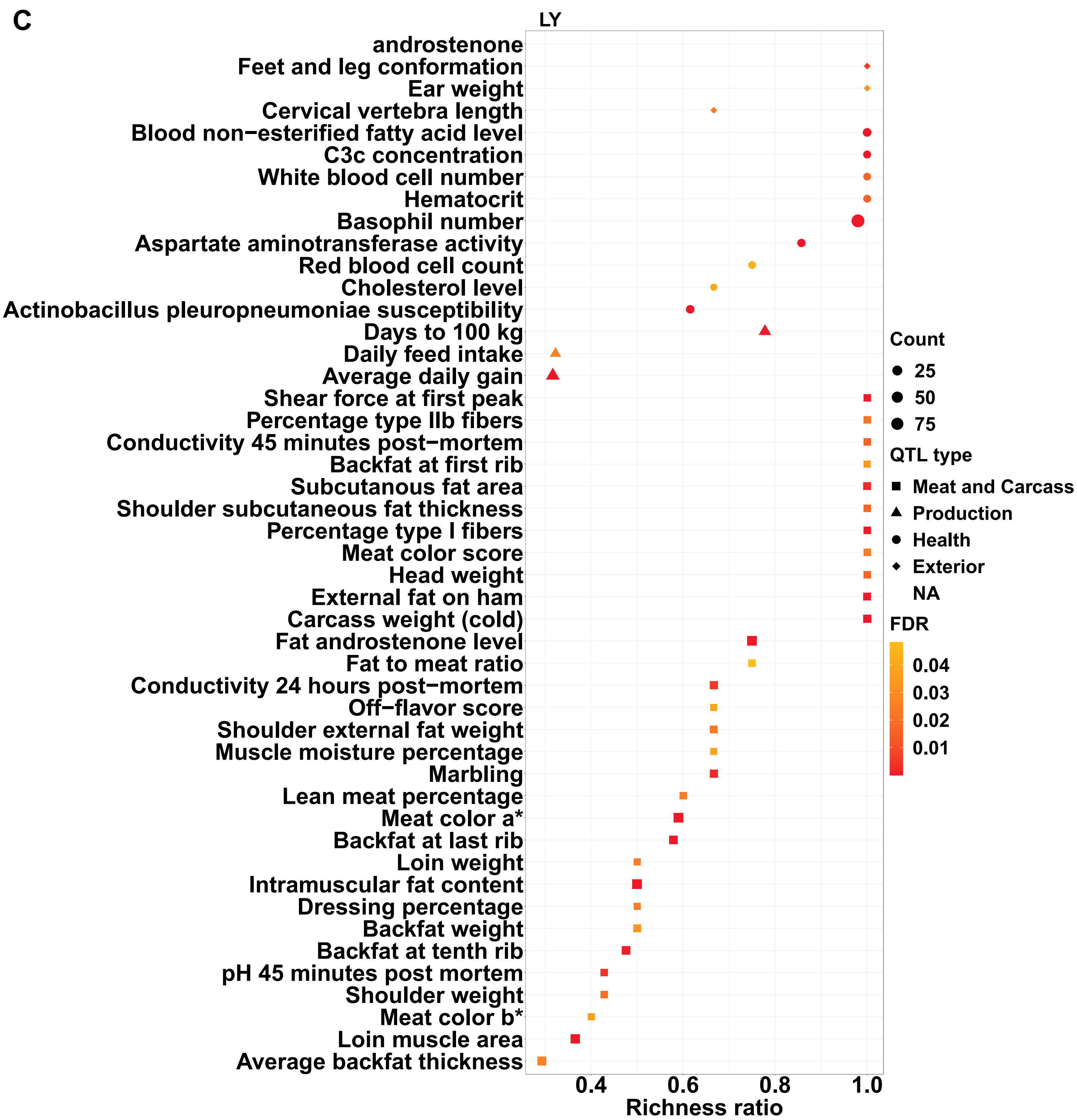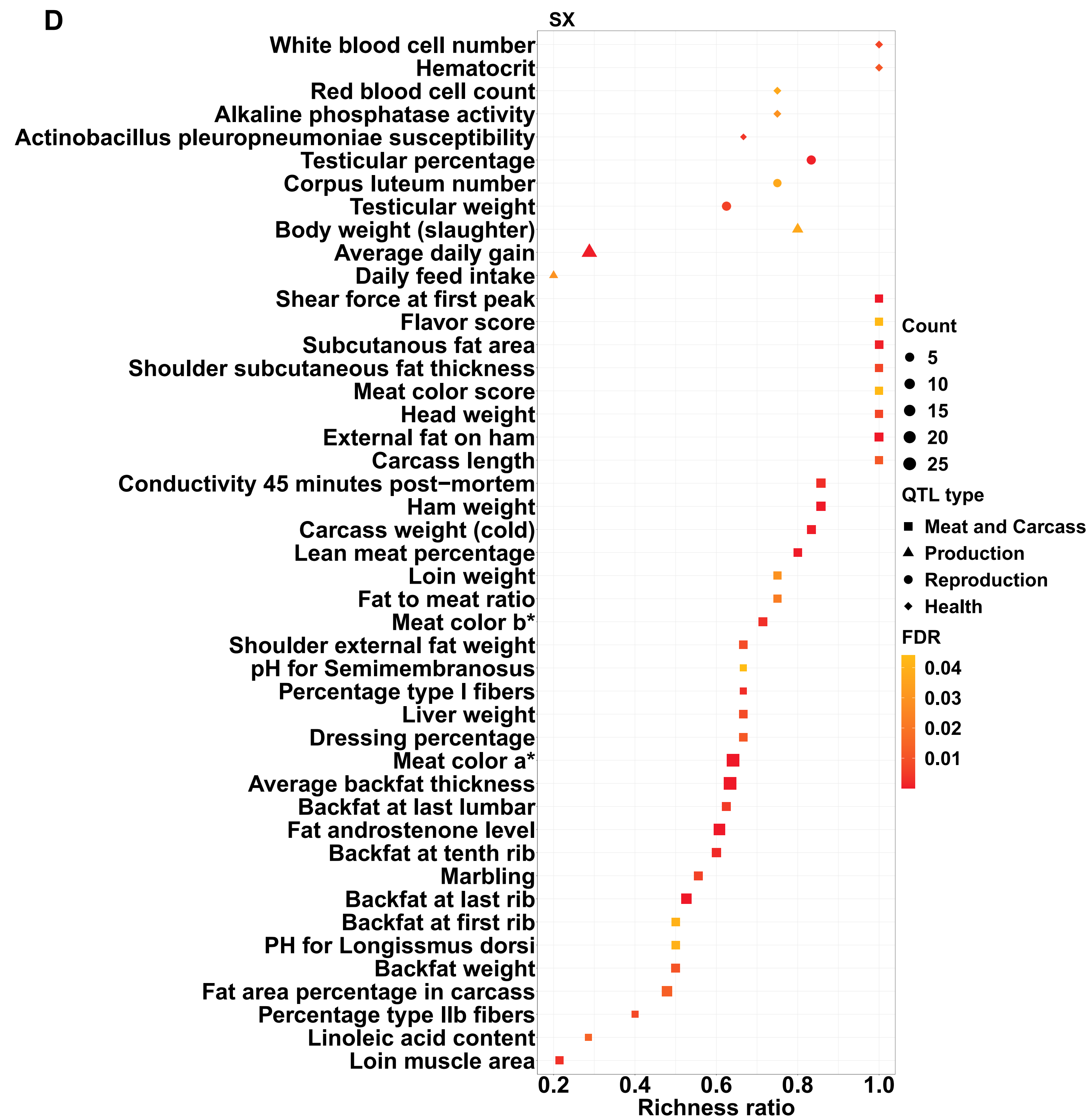

Supplement: Supplementary data 1 [file mmc1.pdf]

**A**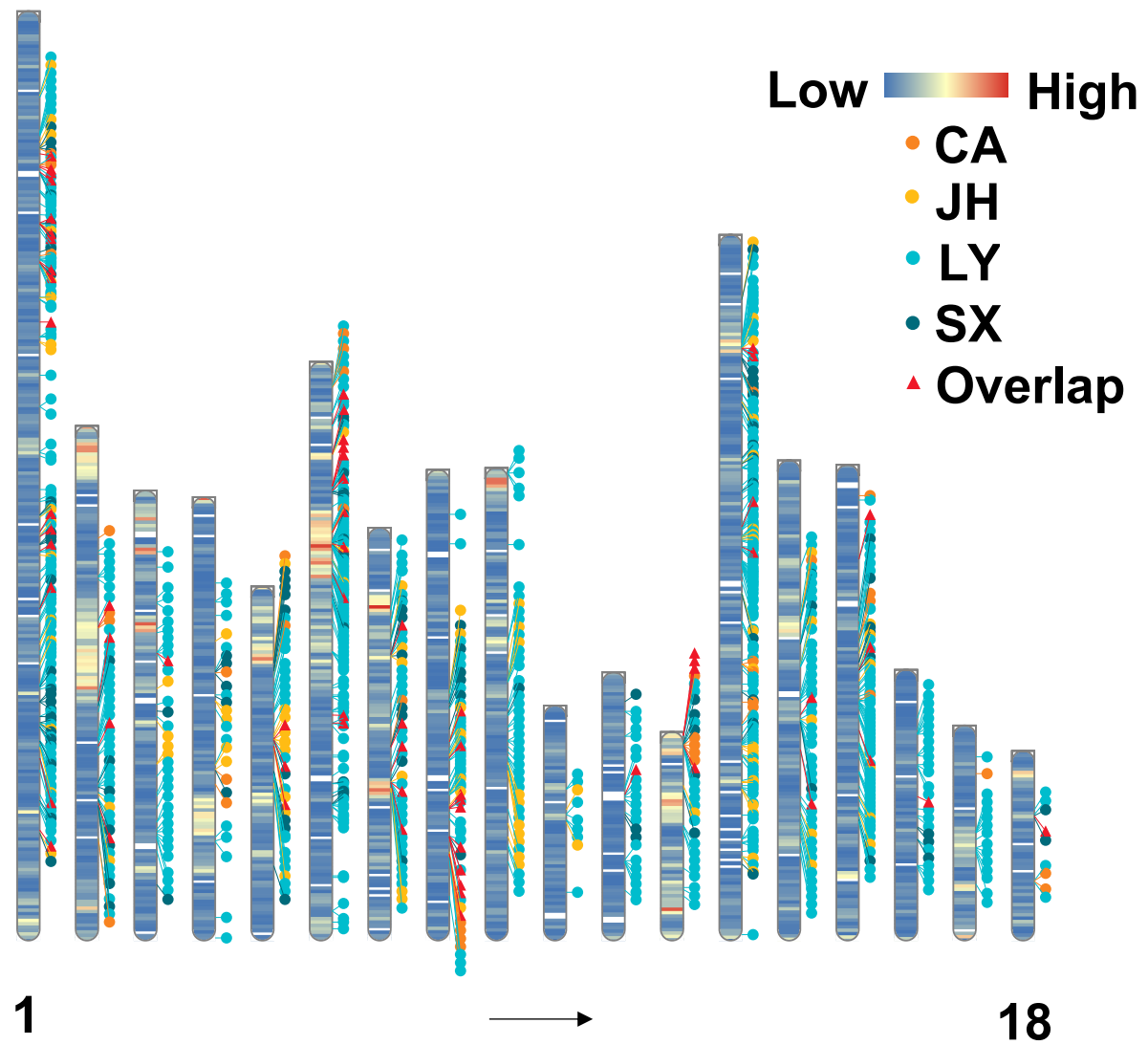**B**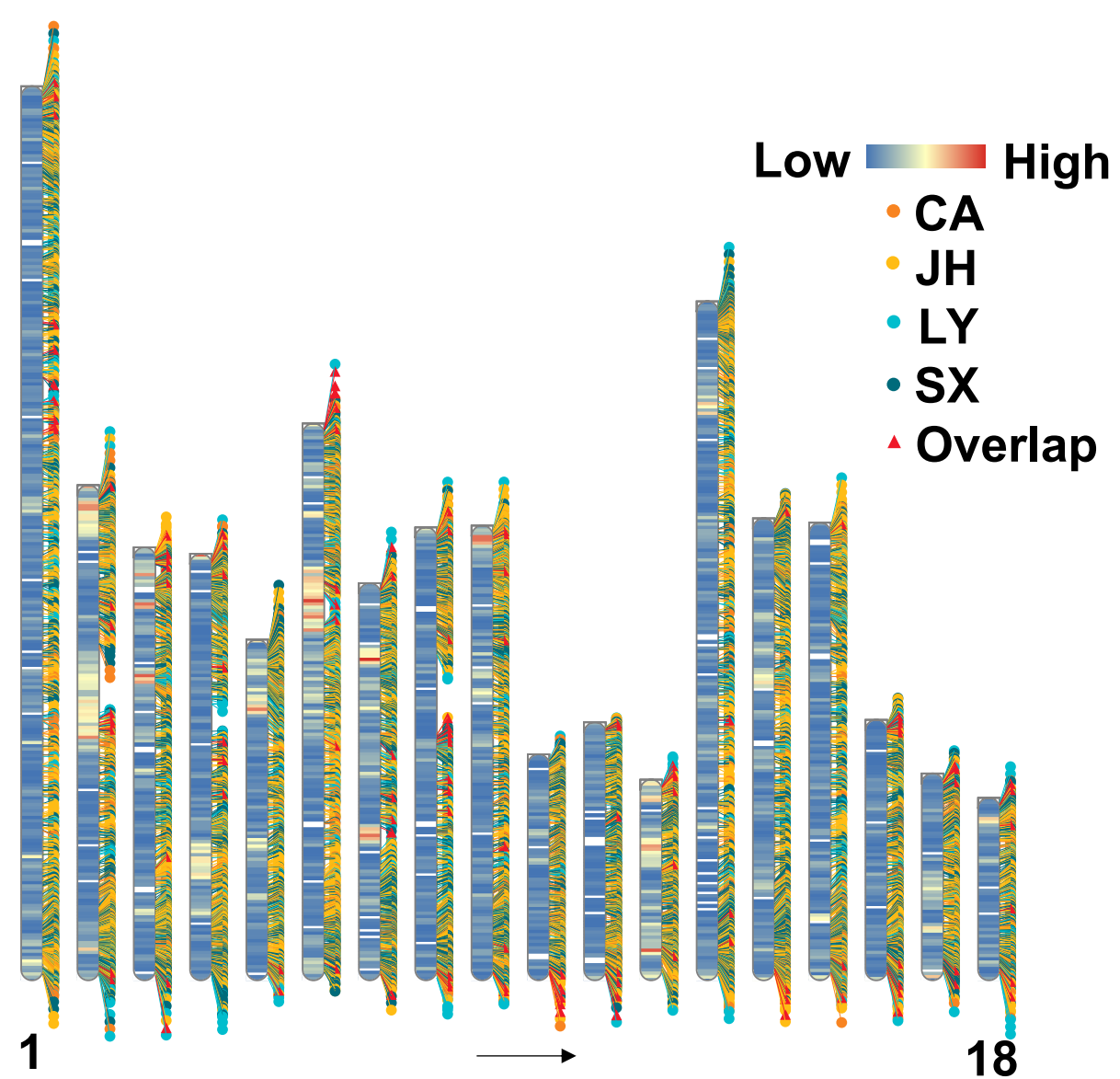

Supplement: Supplementary data 2 [file mmc2.pdf]

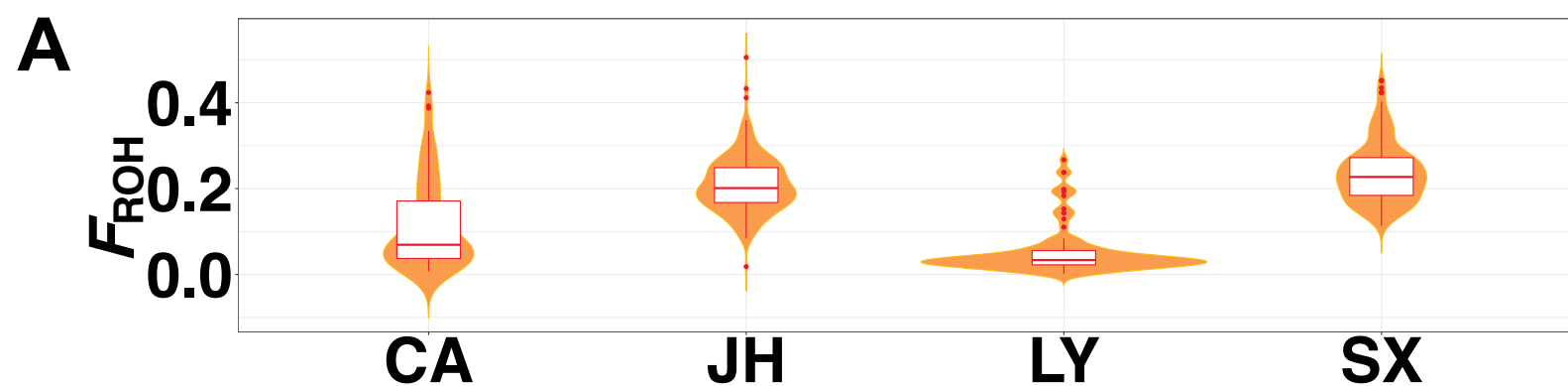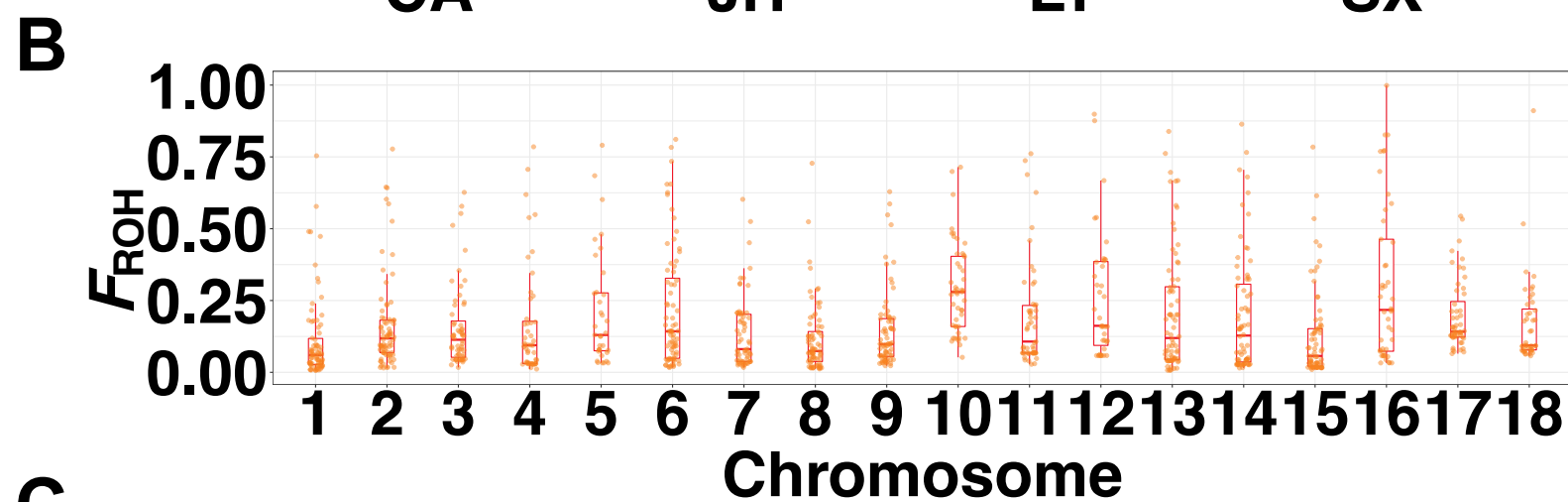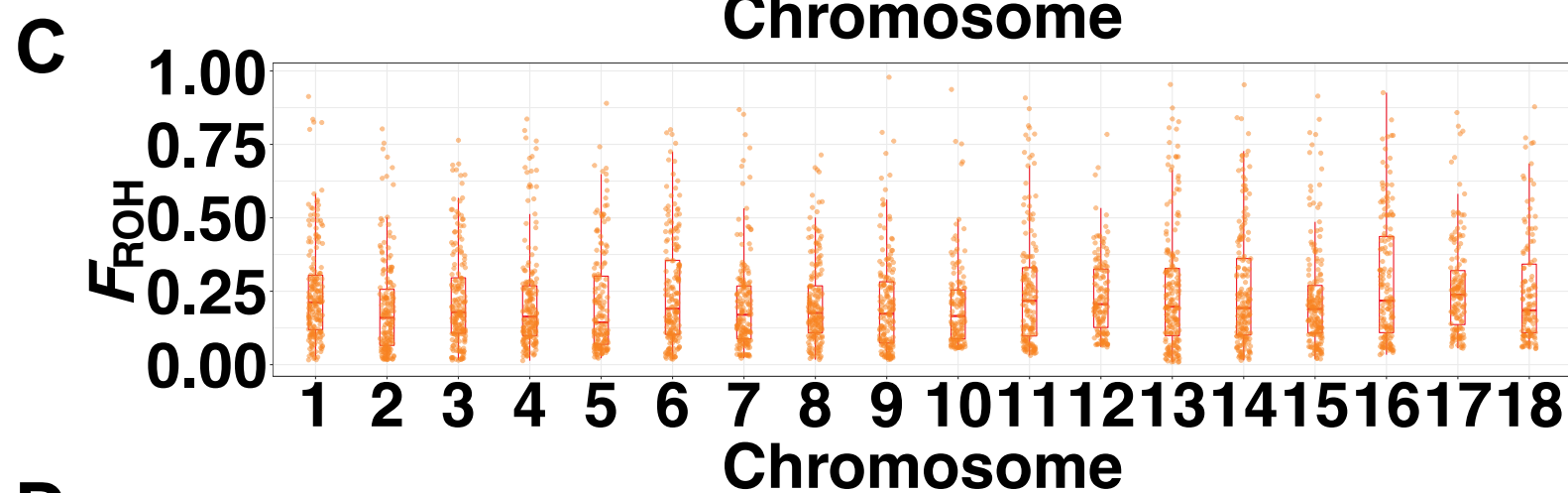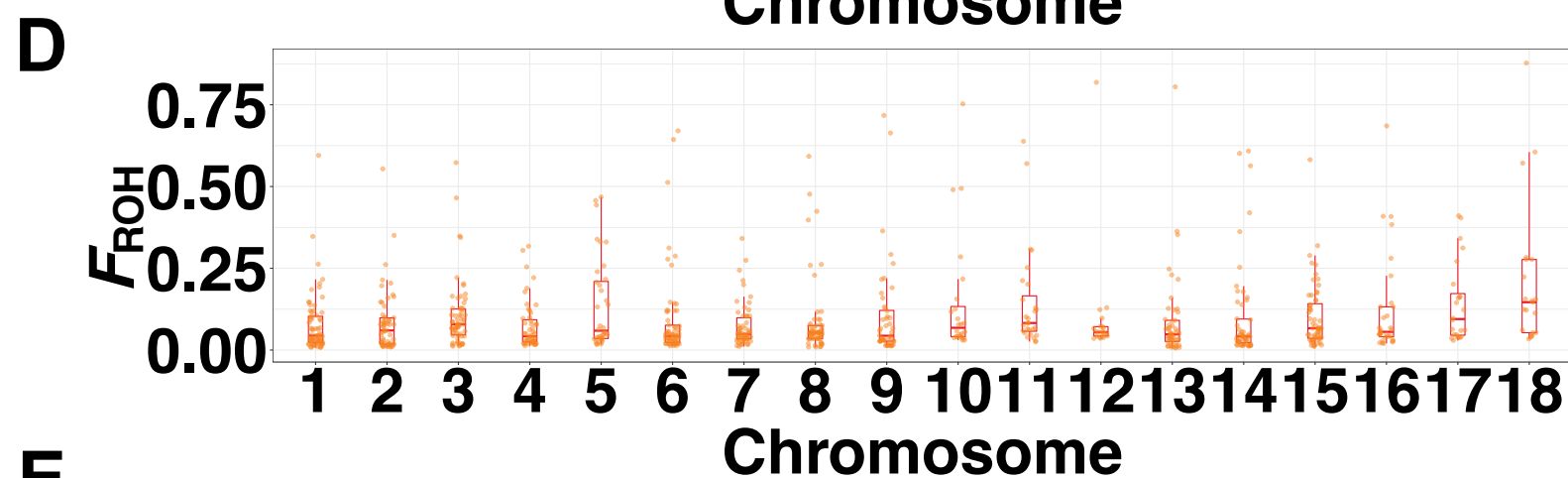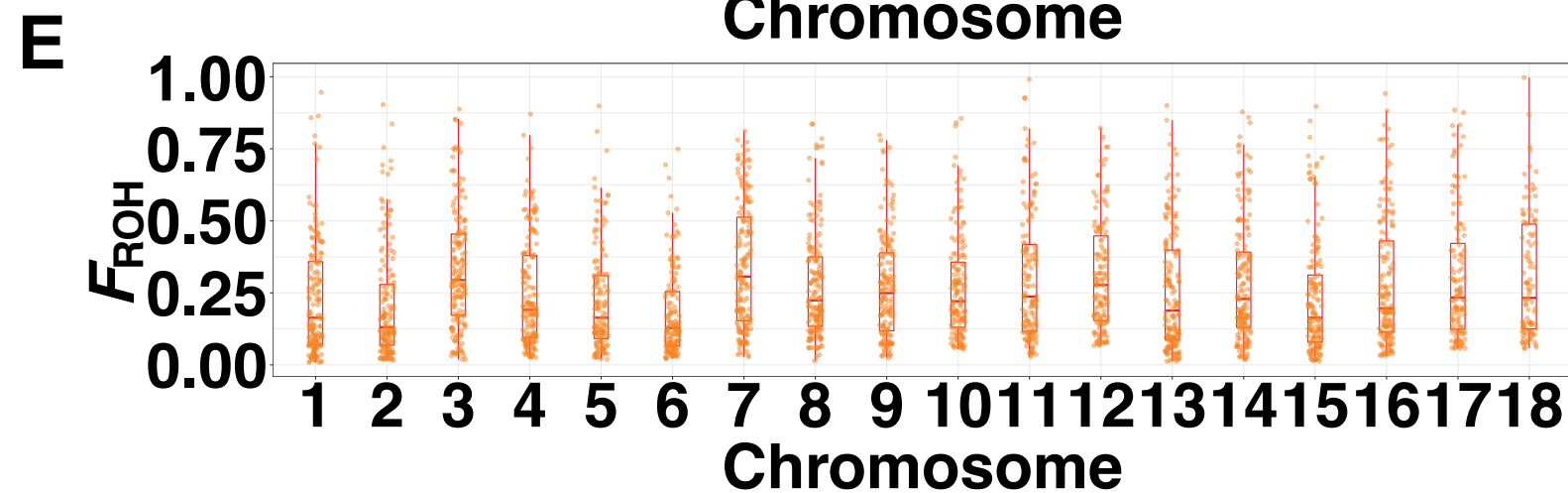

Supplement: Supplementary data 3 [file mmc3.pdf]

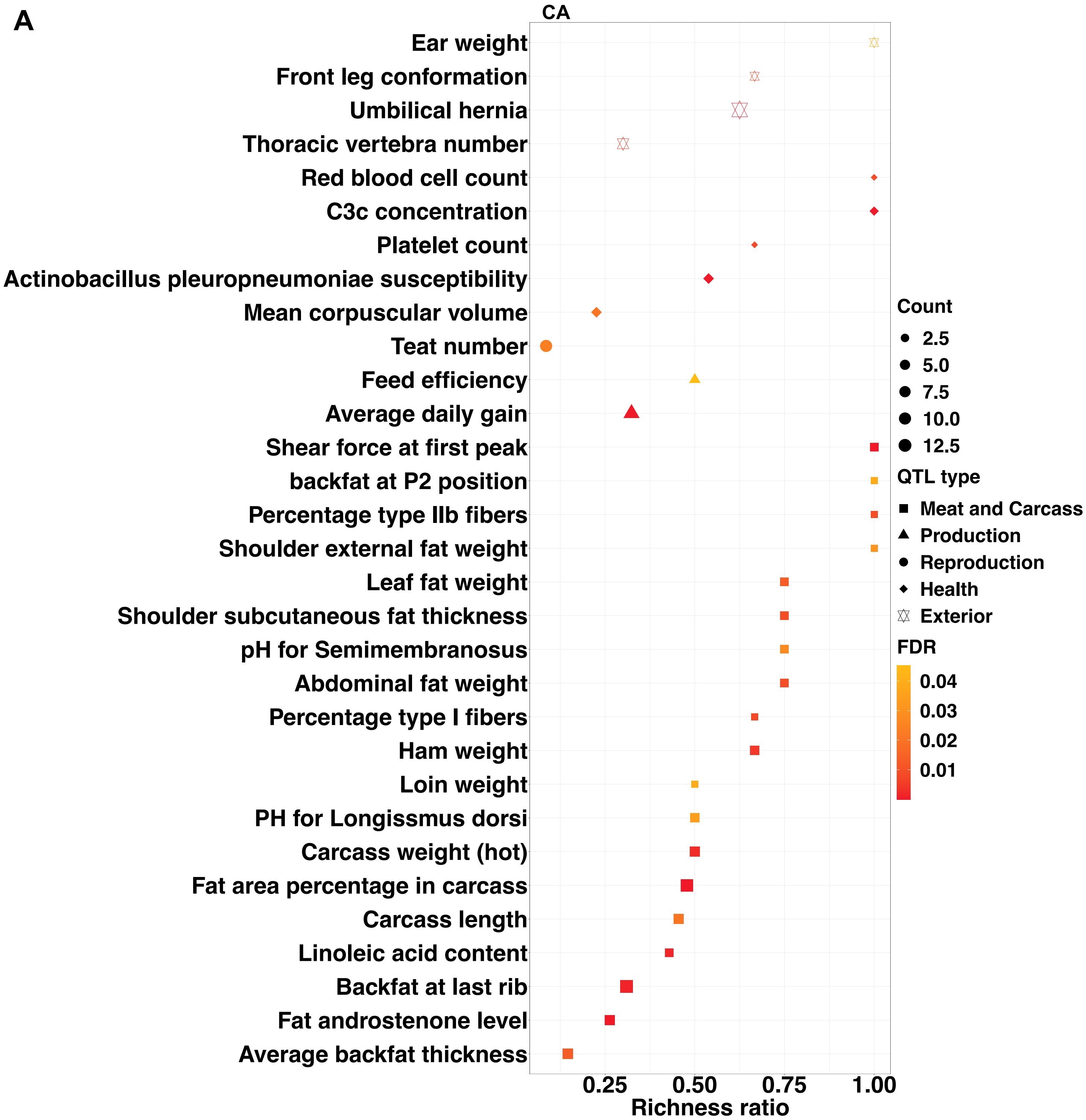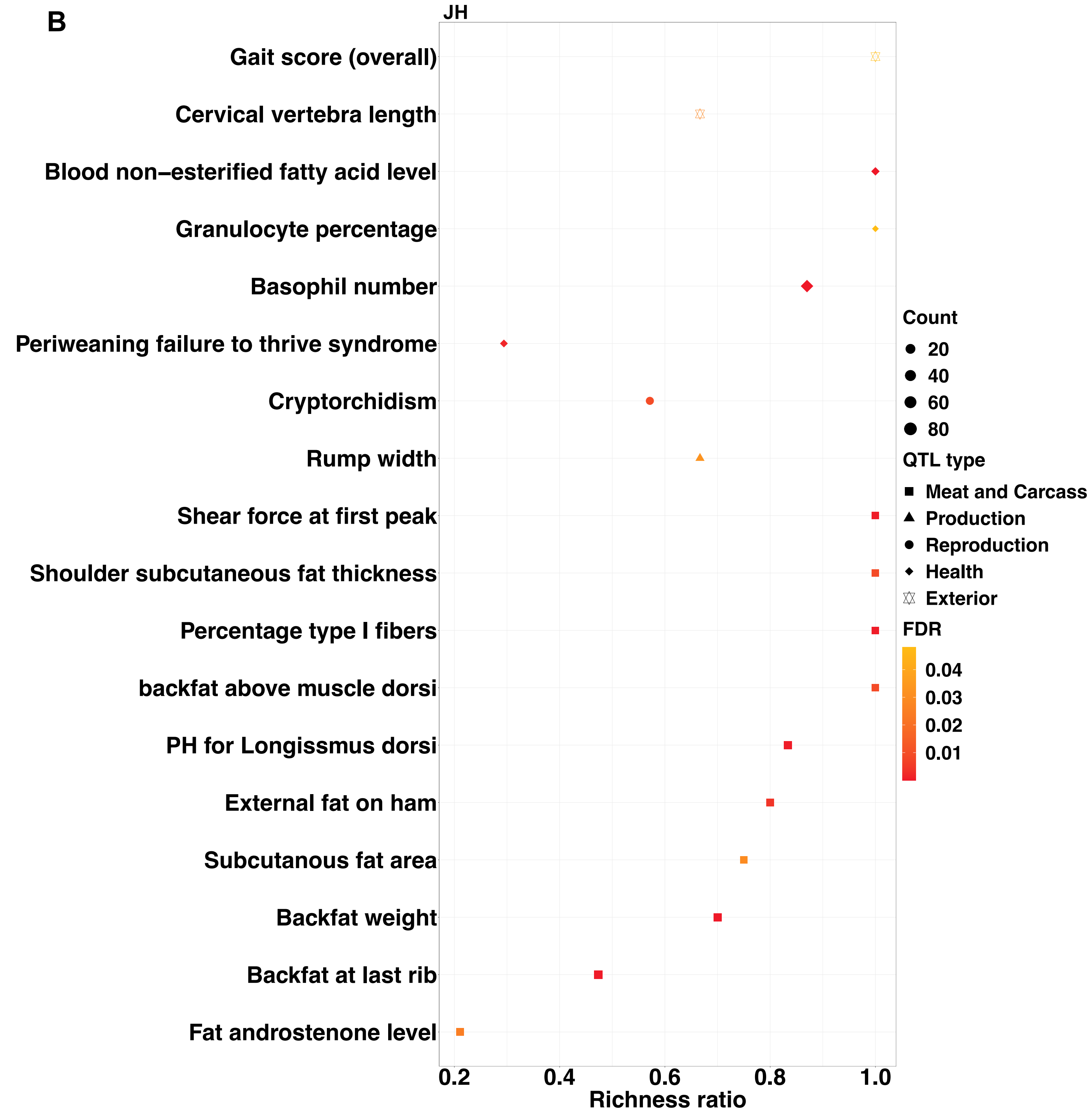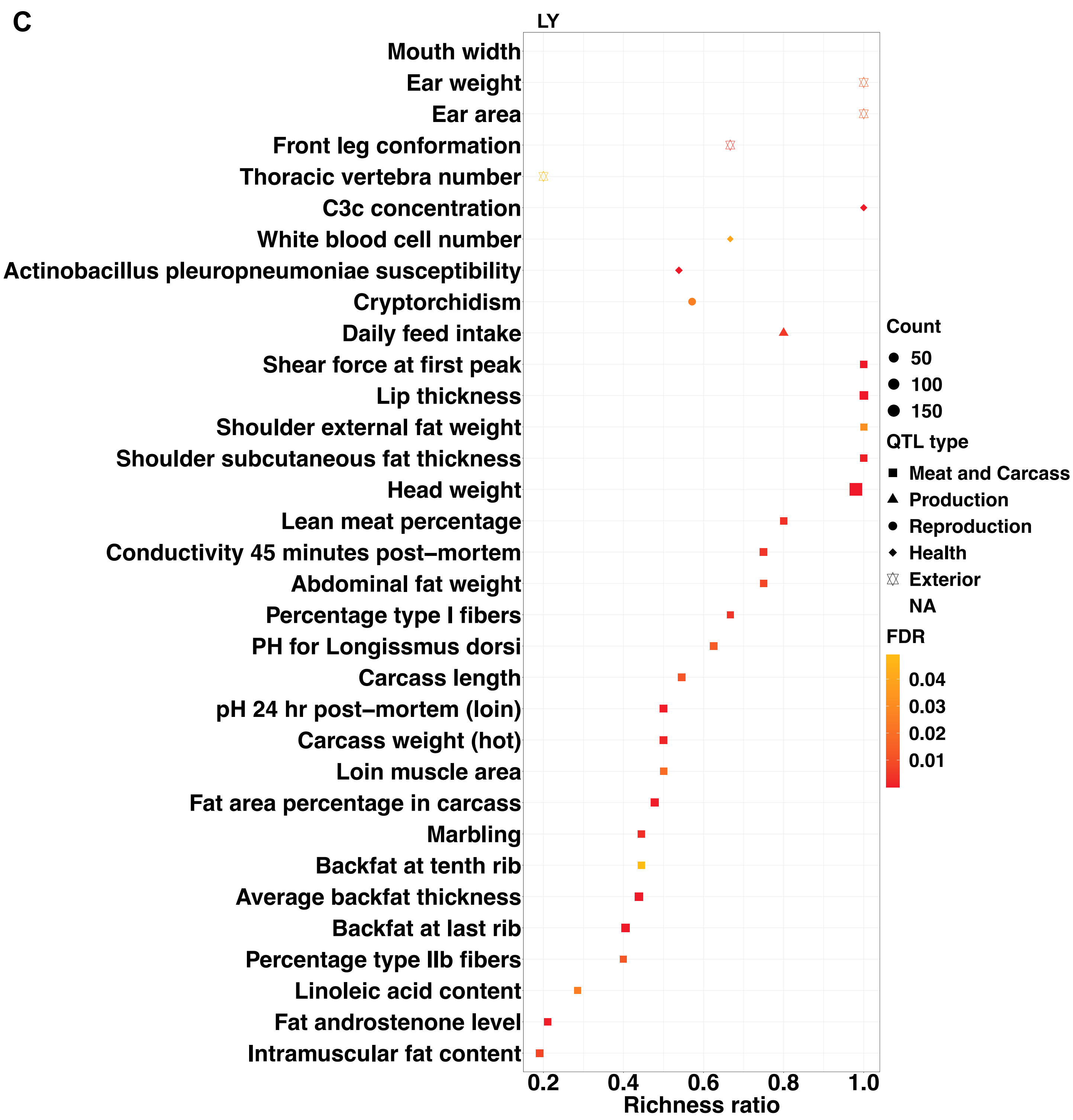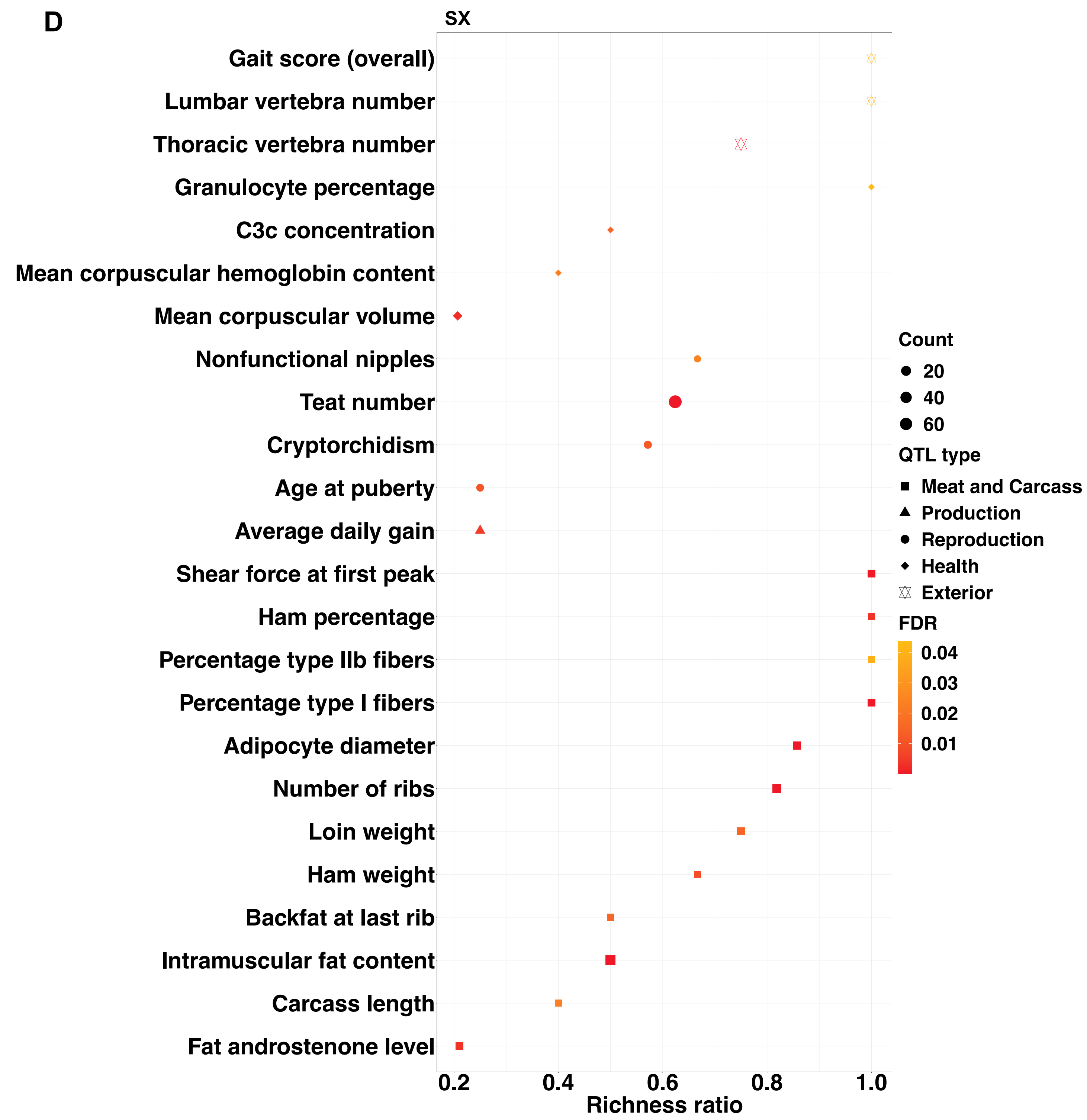

Supplement: Supplementary data 4 [file mmc4.pdf]

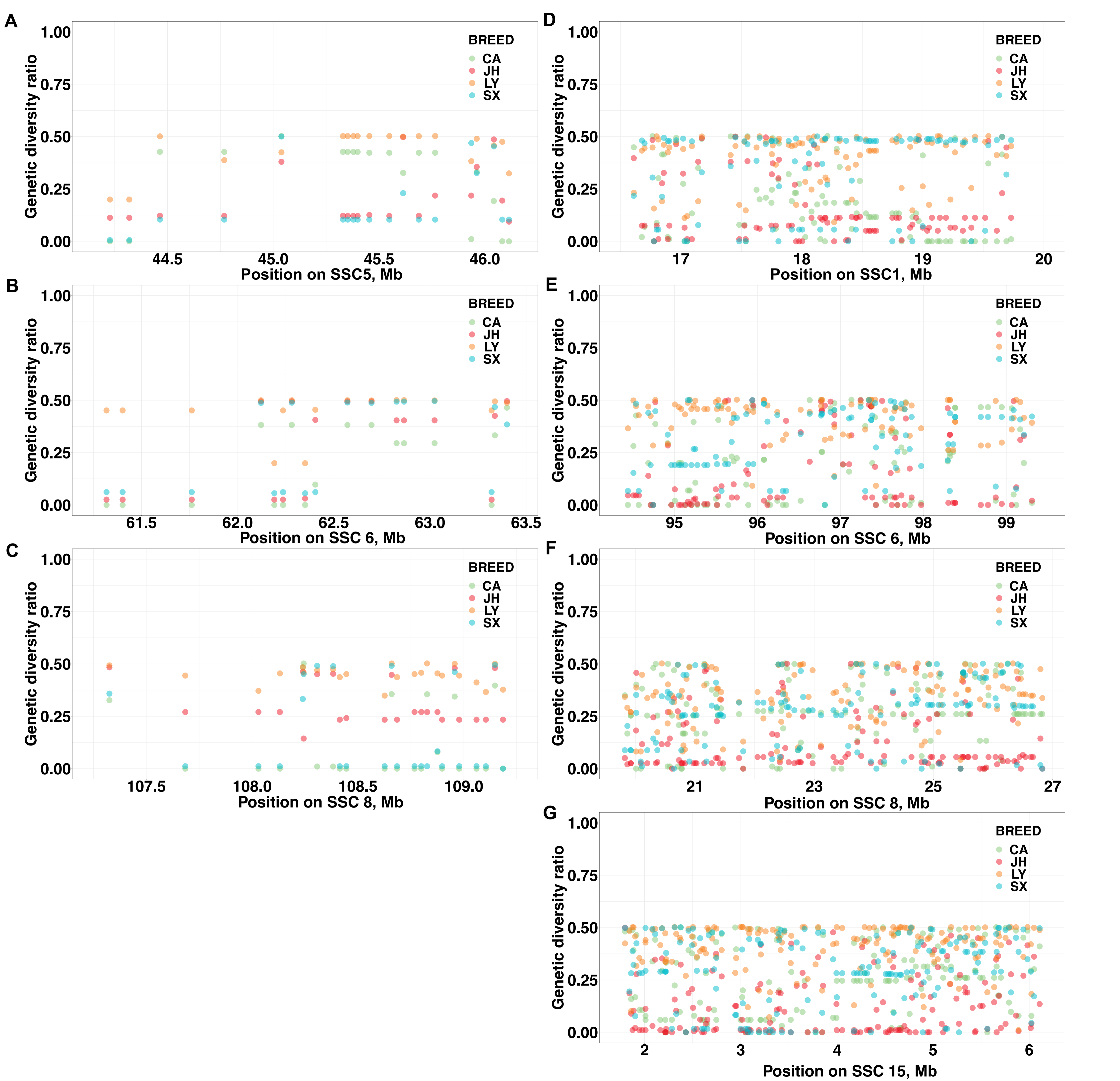

Supplement: Supplementary data 5 [file mmc5.pdf]

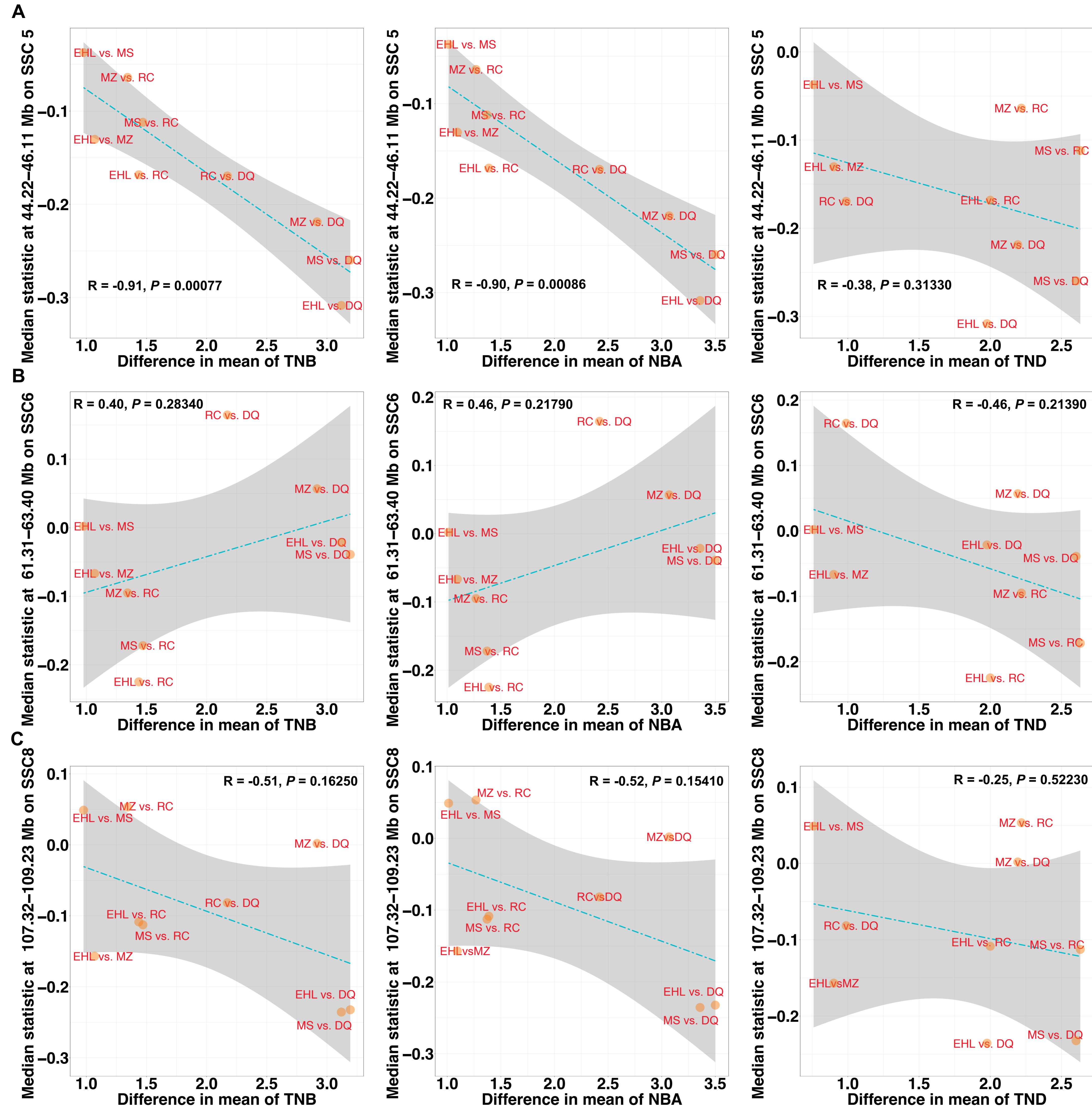

Supplement: Supplementary data 6 [file mmc6.pdf]

A

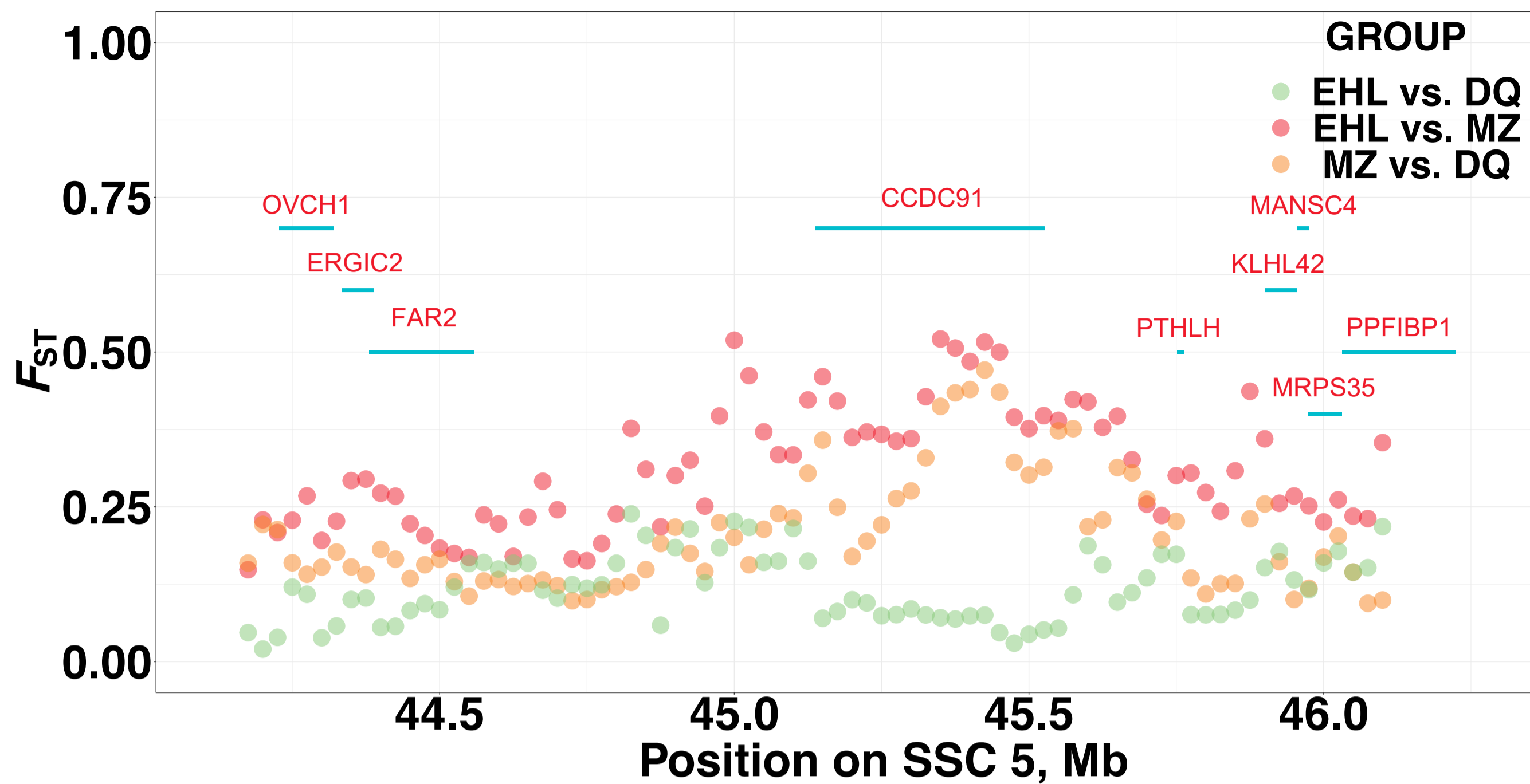

B

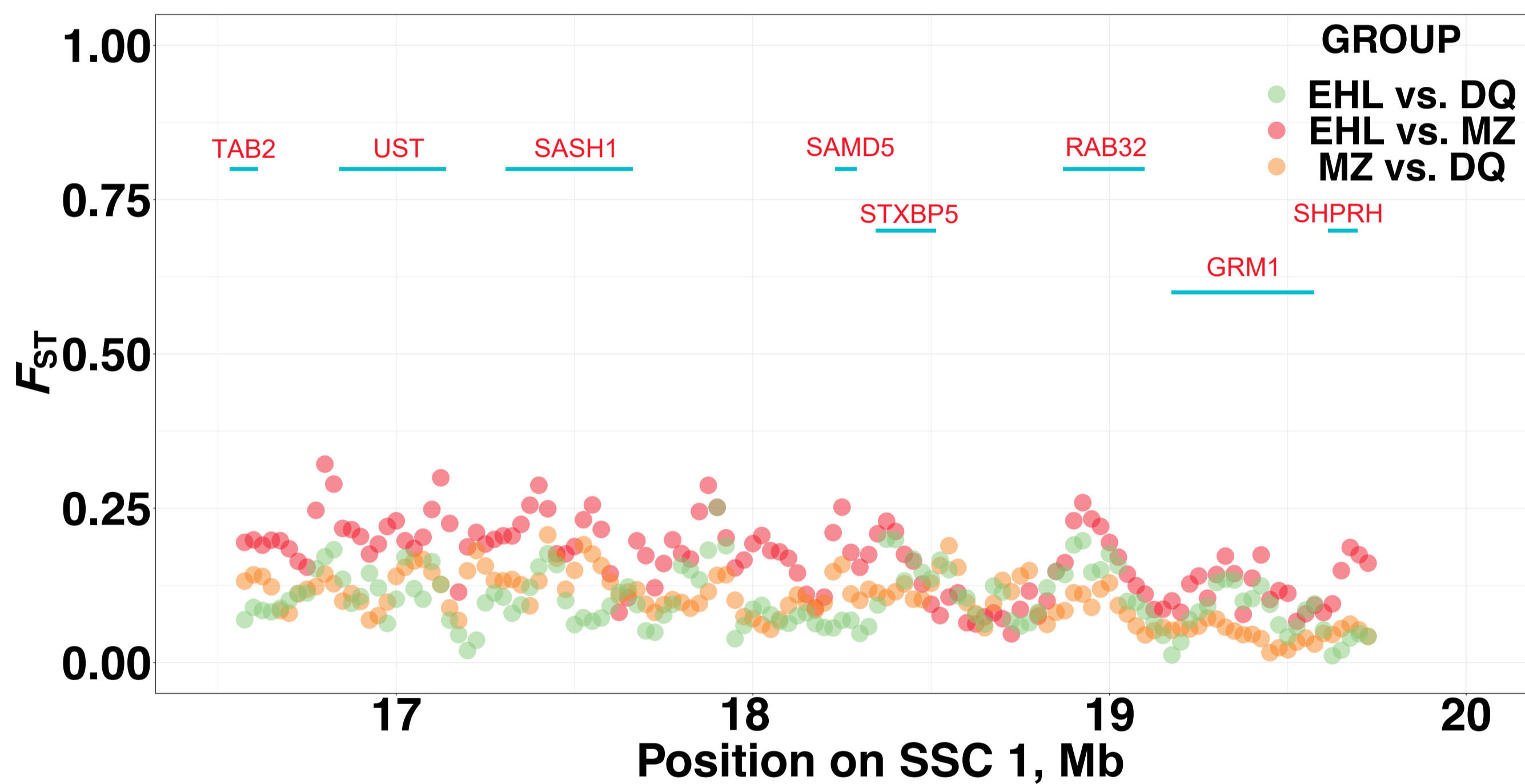

C

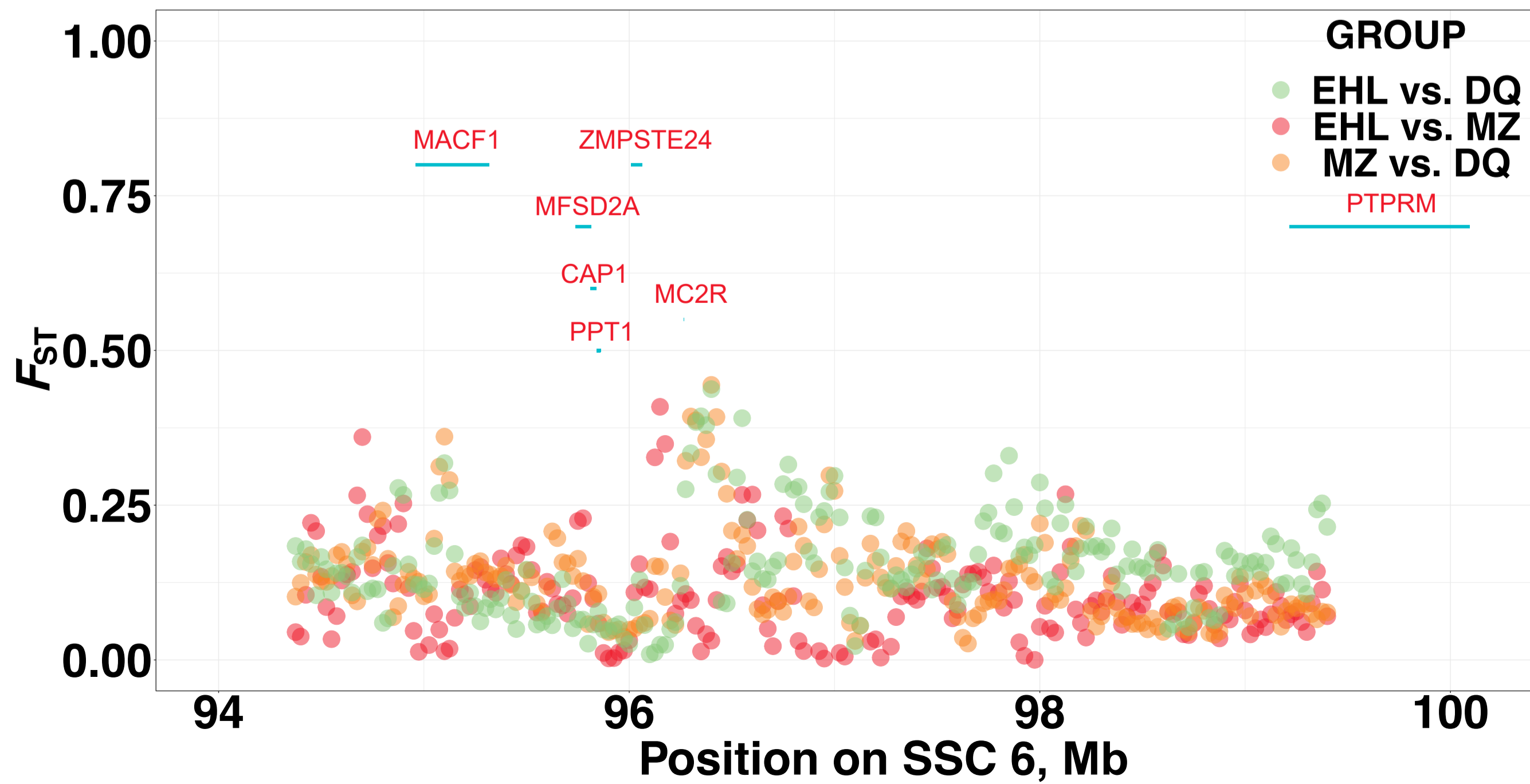

Supplement: Supplementary data 7 [file mmc7.pdf]

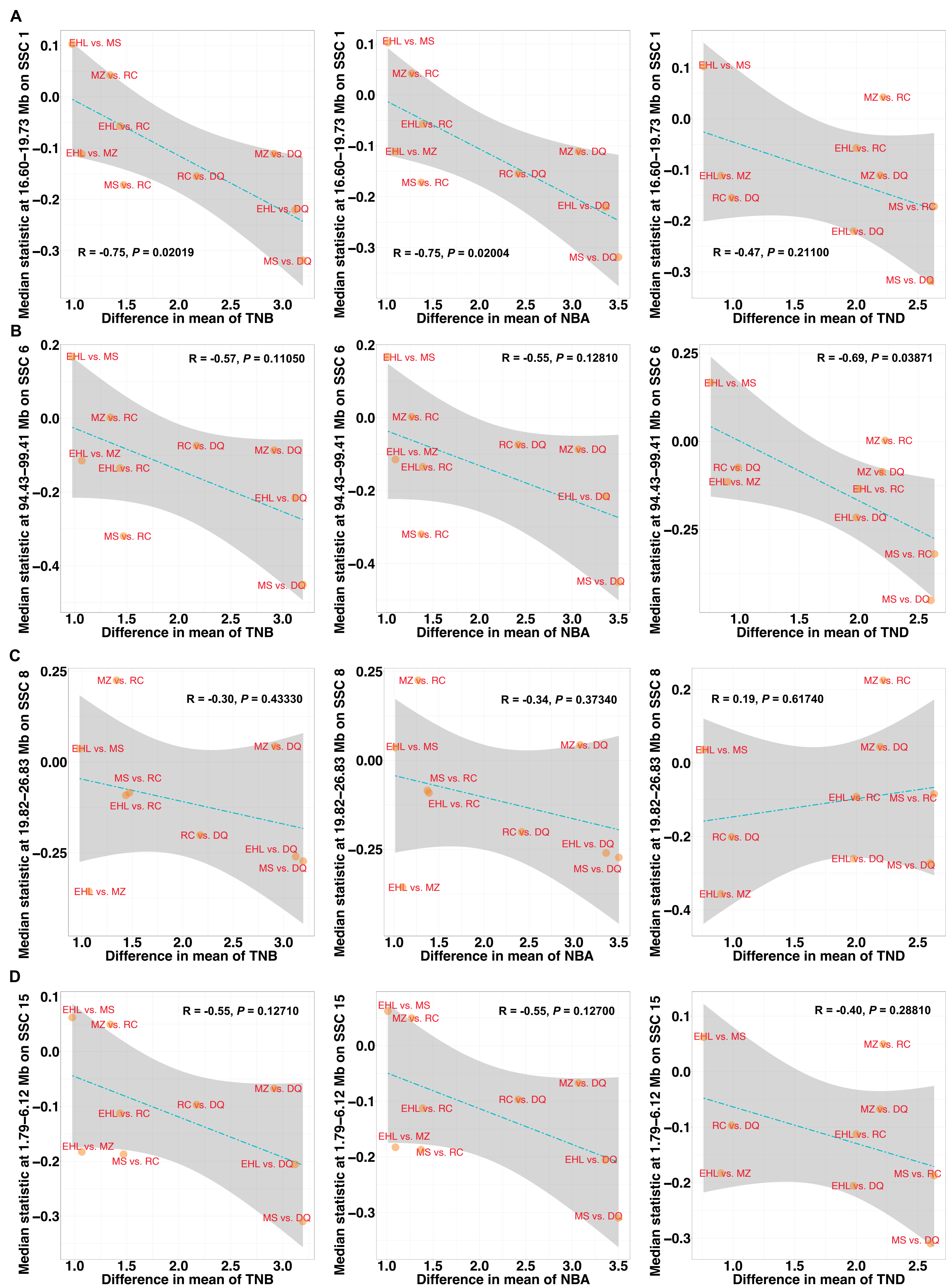

Supplement: Supplementary data 8 [file mmc8.pdf]

**A**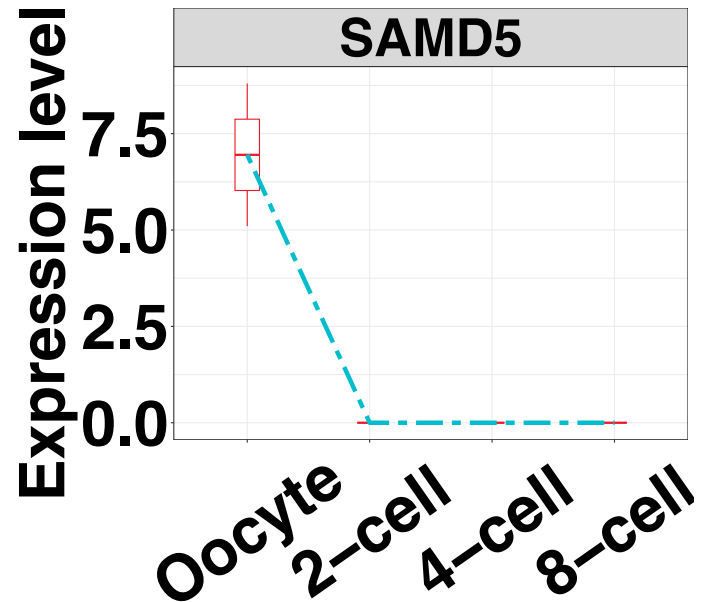**B**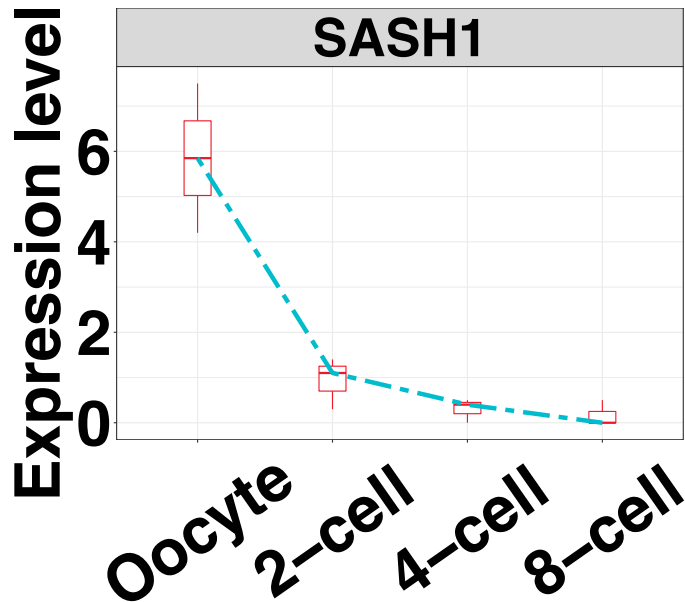

Supplement: Supplementary data 9 [file mmc9.pdf]

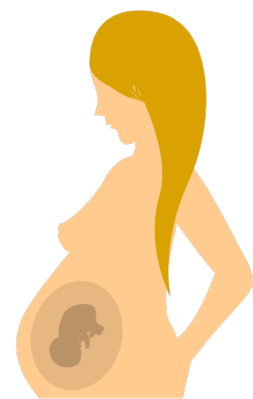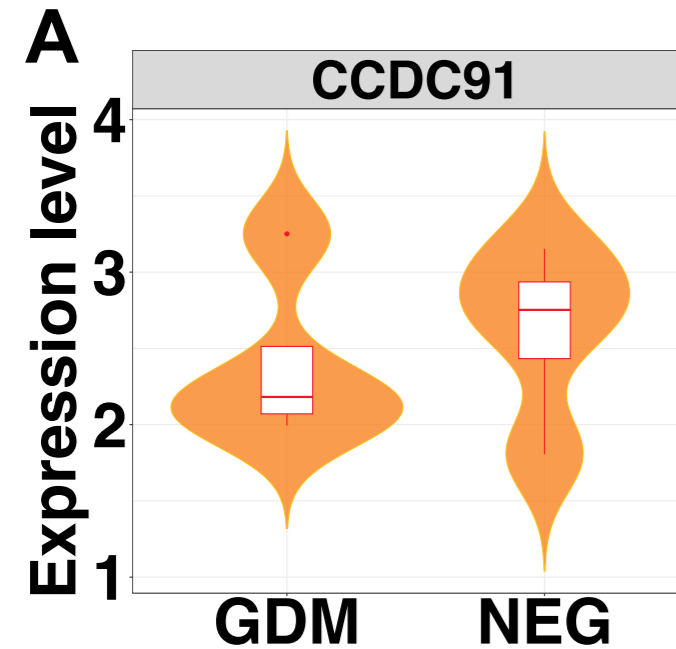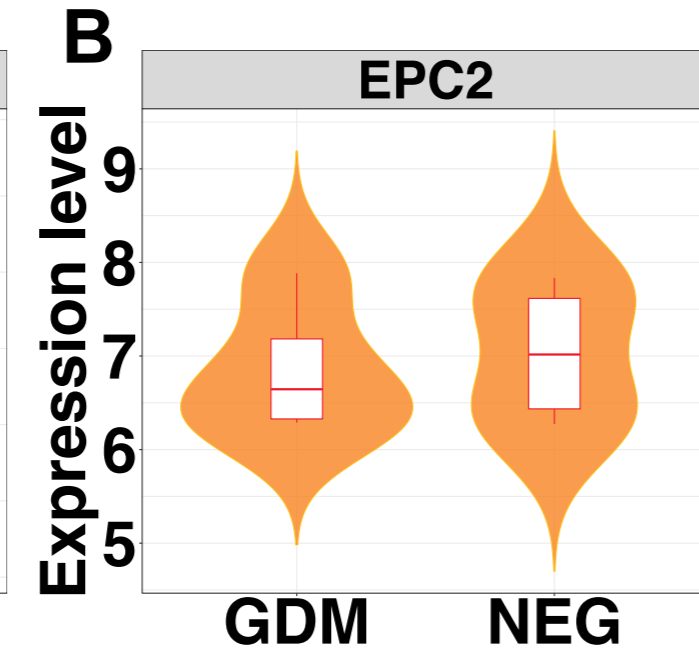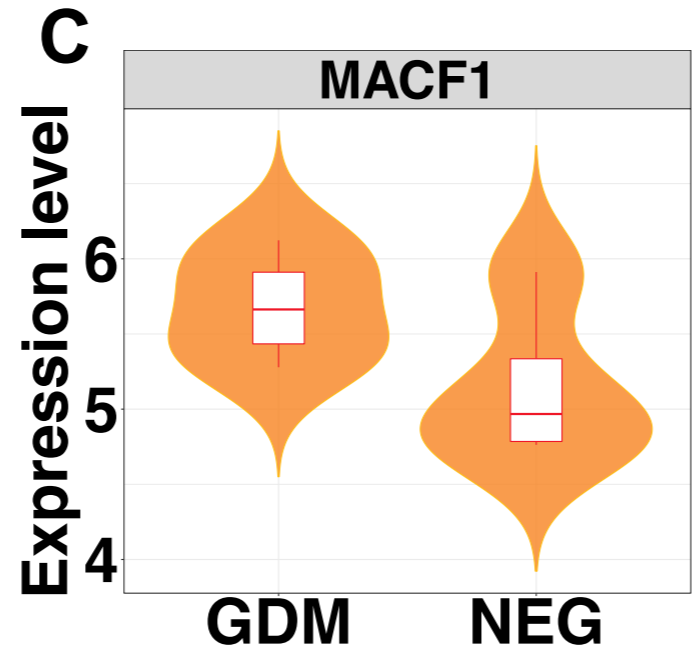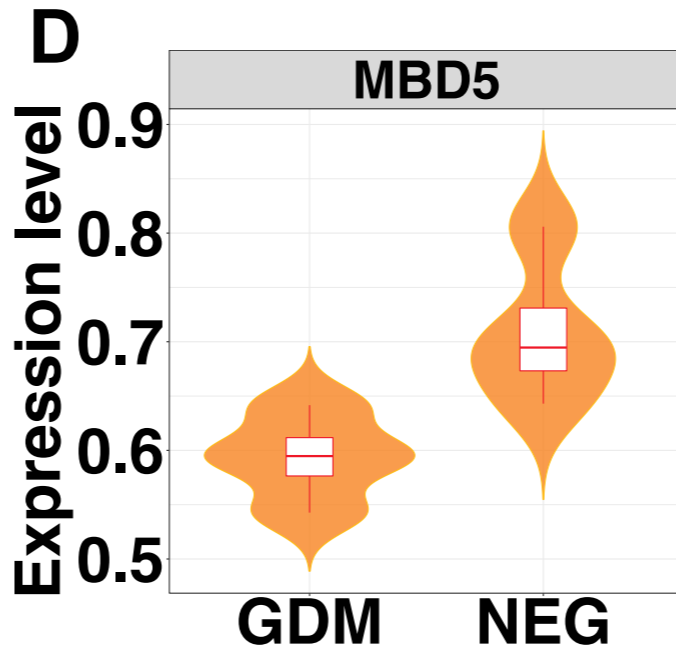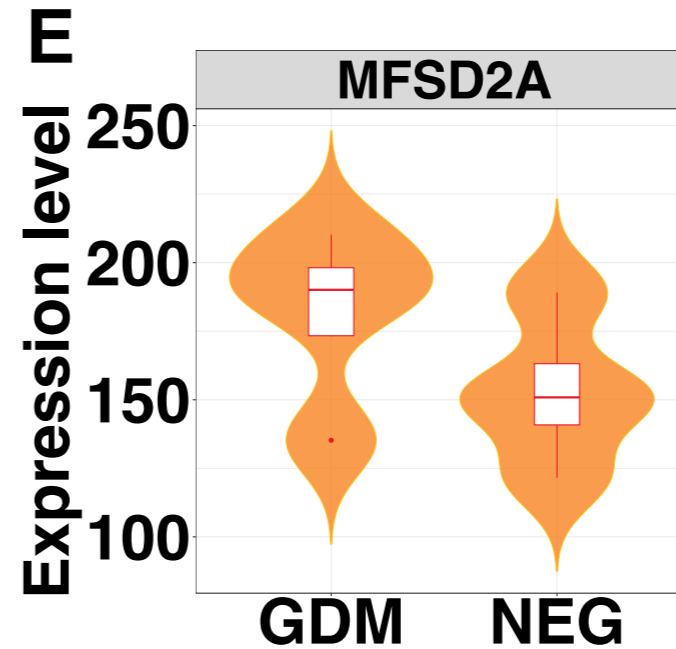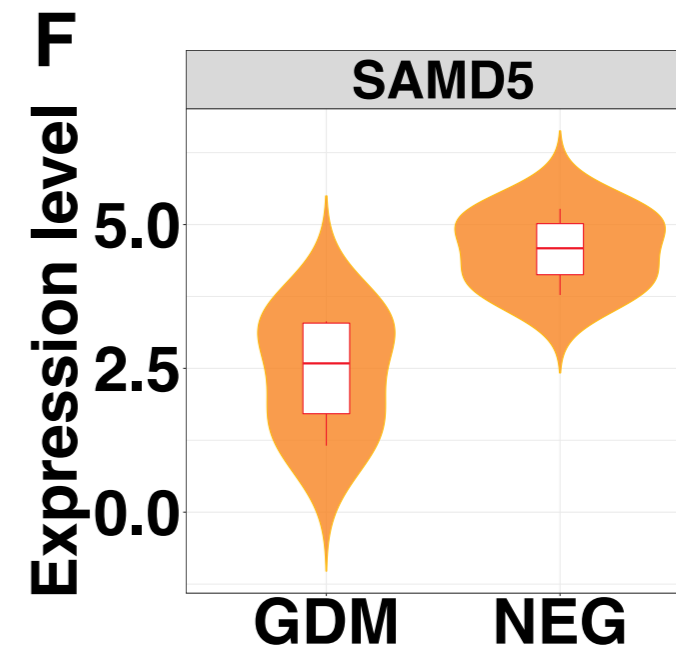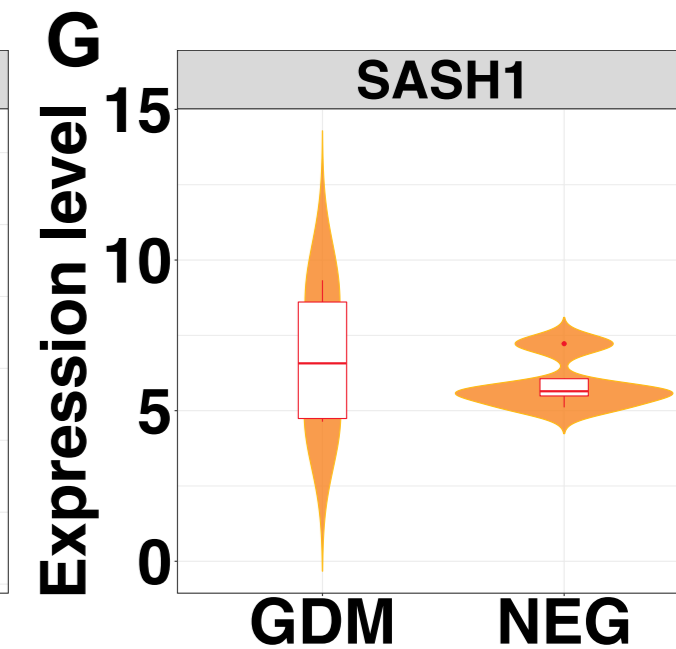

Supplement: Supplementary data 10 [file mmc10.pdf]
